# Functional Characterization of Cytochromes P450 Linked to Herbicide Detoxification and Selectivity in Winter Wheat and the Problem Competing Weed Blackgrass

**DOI:** 10.1021/acsomega.4c11069

**Published:** 2025-03-21

**Authors:** Alina Goldberg-Cavalleri, Sara Franco-Ortega, Stewart Brown, Andrew Walker, Blandine Rougemont, John Sinclair, Melissa Brazier-Hicks, Richard Dale, Nawaporn Onkokesung, Robert Edwards

**Affiliations:** † School of Natural and Environmental Sciences, Newcastle University, Newcastle upon Tyne NE1 7RU, U.K.; ‡ Syngenta, Jealott’s Hill, Bracknell, Berkshire, Warfield RG42 6EY, U.K.

## Abstract

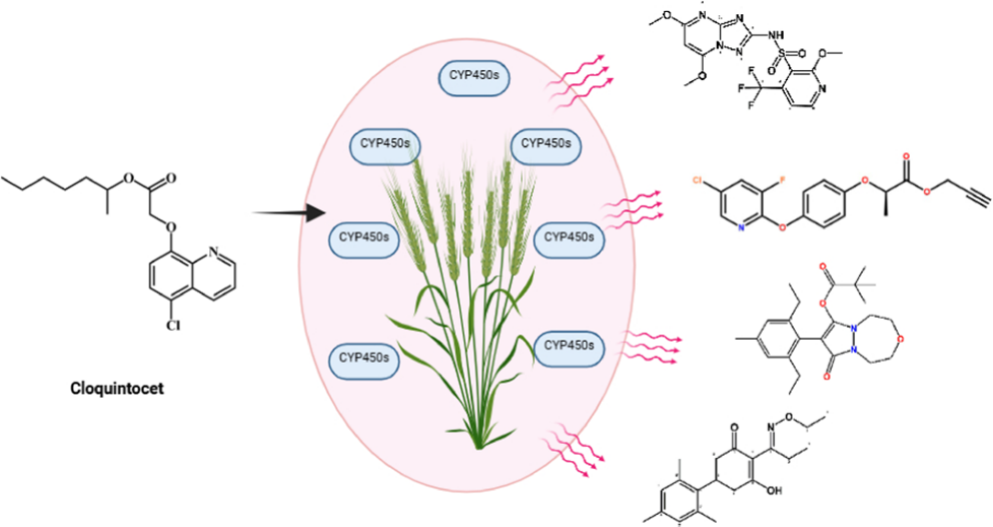

The selective chemical control of wild grasses in wheat is primarily determined by the relative rates of herbicide metabolism, with the superfamily of cytochromes P450 (CYPs) playing a major role in catalyzing phase 1 detoxification reactions. This selectivity is enhanced by herbicide safeners, which induce CYP expression in cereals, or challenged by the evolution of nontarget site resistance (NTSR) in weeds such as blackgrass. Using transcriptomics, proteomics, and functional expression in recombinant yeast, CYPs linked to safener treatment and NTSR have been characterized in wheat and blackgrass. Safener treatment resulted in the induction of 13 families of CYPs in wheat and 5 in blackgrass, with CYP71, CYP72, CYP76, and CYP81 members active toward selective herbicides in the crop. Based on their expression and functional activities, three inducible *Ta*CYP81s were shown to have major roles in safening in wheat. In contrast, a single *Am*CYP81 that was enhanced by NTSR, but not by safening, was found to dominate herbicide detoxification in blackgrass.

## Introduction

Selective weed control using herbicides is a cornerstone of modern arable agriculture, protecting cereal crops from weed competition, which would otherwise result in up to 34% losses in yield on a global scale.^1^ Controlling wild grasses in cereals is a particular challenge, as similarities in the physiology of the crop and weed mean there are no obvious differences in the herbicide mode of action to exploit. Instead, the primary basis for selective chemical control in nongenetically modified crops relies on the differential rates of herbicide detoxification, with domesticated cereals metabolizing the active ingredient more rapidly than competing wild grasses.^2,3^ Herbicide detoxification in both crops and weeds occurs through a phased series of reactions, with the primary biotransformations typically involving oxidations to produce metabolites containing functional groups (phase 1), which can then undergo bioconjugation (phase 2) and transport (phase 3), prior to further processing and sequestration.^4−6^ Most commonly, these phase 1 oxidative reactions are catalyzed by cytochromes P450 (CYPs), a group of membrane-associated monooxygenases that utilize the electrons generated from NADPH through the action of a cognate CYP reductase, together with molecular oxygen, to catalyze hydroxylations, epoxidations, dealkylations, and bond cleavages on a diverse range of chemistries.^7−9^ Being primary effectors of herbicide metabolism, CYPs are often considered the rate-limiting step in herbicide biotransformation and, therefore, integrally linked to defining selectivity between crops and weeds.^9−11^

**Figure 1:**
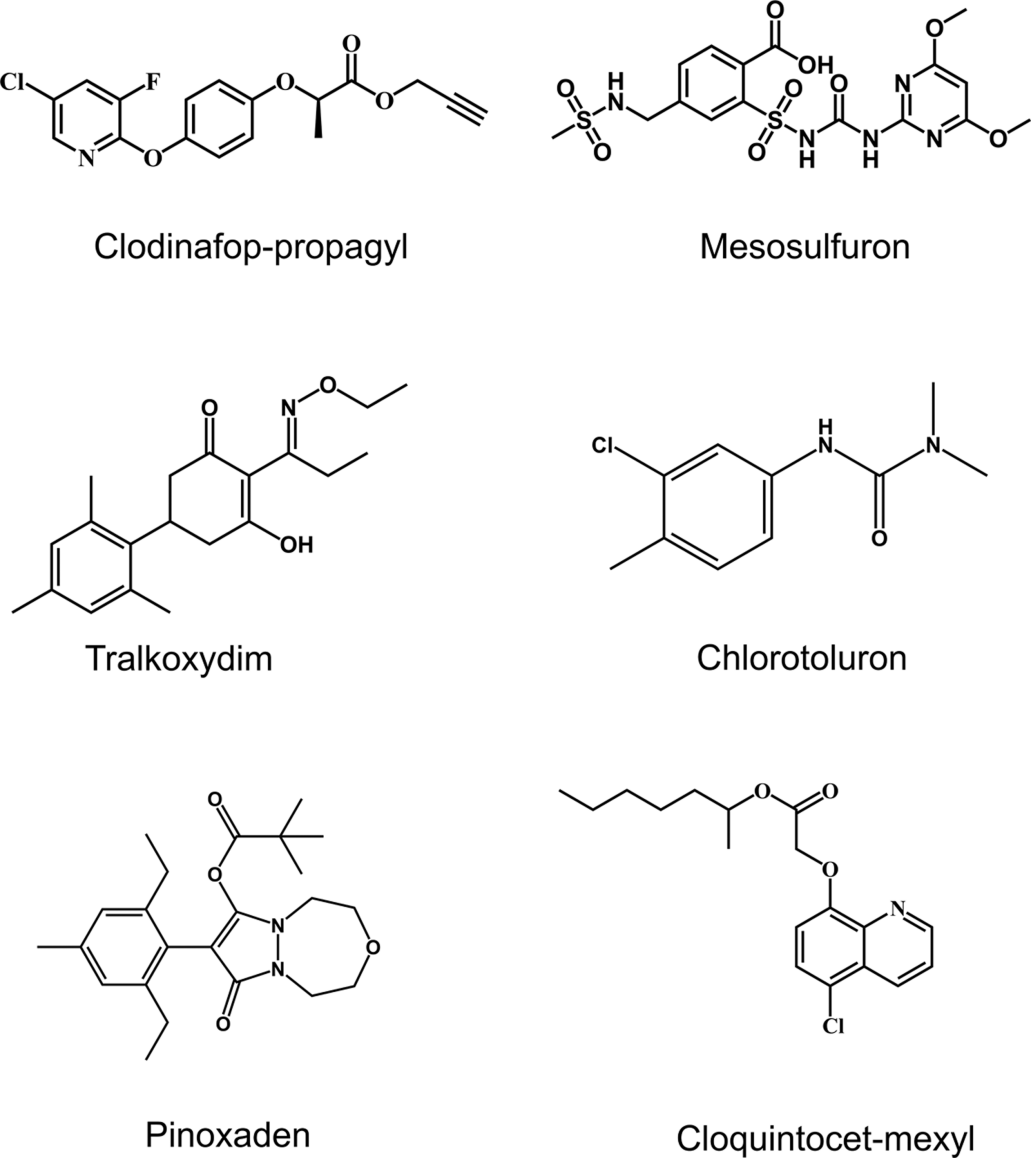
Structures of herbicides and the safer cloquintocet-mexyl used in this study.

Herbicide selectivity in winter wheat (*Triticum aestivum* L.), the major arable crop in the United Kingdom, is of considerable current interest, with respective crop production challenged by competition from several wild grasses, with blackgrass (*Alopecurus myosuroides* L.) currently ranked as the most problematic.^12−14^ The selective control of blackgrass is highly dependent on the use of postemergence herbicides, notably aryloxyphenoxypropionates (FOPs, e.g., clodinafop-propargyl), cyclohexanediones (DIMs, e.g., tralkoxydim), phenylpyrazoles (e.g., pinoxaden), sulfonylureas (SUs, e.g., mesosulfuron), and historically phenylureas (PUs, e.g., chlorotoluron). The structures of herbicides from each of these classes, which have been used in this study, are presented in Figure 1. Over the years, a body of data has demonstrated the importance of metabolism in defining the selectivity of these herbicides in wheat vs several wild grasses, including the problem weed blackgrass.^15,17^ To enhance the margins for selective weed control in wheat, the SU, DIM, and FOP classes of herbicides are typically coapplied with herbicide safeners, such as cloquintocet-mexyl (Figure 1). Safeners enhance selectivity by accelerating the metabolism of herbicides in cereal crops by inducing the expression of genes encoding proteins involved in detoxification, notably CYPs.^3,17−19^ Because of the importance of safening in detoxification and selectivity, the identification of the associated inducible genes has proven to be a useful means of identifying key enzymes involved in herbicide metabolism, including CYPs, in several cereal crops.^19−21^

With respect to blackgrass control in the UK, the last 4 decades have seen the widespread emergence of populations of this weed showing resistance to all the classes of postemergence herbicides used in wheat.^13,14^ Such resistance has evolved through a number of mechanisms, the most problematic being nontarget site resistance (NTSR), which can impart tolerance to multiple classes of selective herbicides.^4−6,22^ NTSR is a complex, multigenic, and quantitative trait, with affected populations exhibiting different levels of tolerance to FOP, DIM, SU, and PU herbicides. While NTSR involves the activation of multiple cytoprotective pathways,^4−6^ the enhanced expression of herbicide-detoxifying enzymes and associated transporters is classically associated with this class of resistance. Thus, populations of NTSR blackgrass have been shown to metabolize PU,^22^ SU,^23^ FOP,^16,24^ and phenylpyrazole^25^ herbicides more rapidly than the respective herbicide-sensitive (HS) plants. As with safening in the crop, the enhancement in herbicide metabolism in NTSR blackgrass is linked to the elevated expression of detoxifying CYPs.^22,26,27^

In this study, we have characterized the complement of CYPs in wheat and blackgrass that are active in detoxifying selective herbicides. To assist in the determination of critical detoxifying enzymes, we have used safening in wheat and NTSR in the competing weed blackgrass, respectively, to identify up-regulated family members that are associated with altered tolerance to herbicides and are therefore most likely to be the critical CYPs determining selectivity. The approach adopted has been to first use both seedlings and the respective cell cultures for each species, in a combination of global transcriptomics and proteomics, to identify the complement, the “CYPome”, of wheat and blackgrass, respectively. Based on their profile of expression in response to safeners (wheat) or NTSR (blackgrass), as well as their relative abundance *in planta*, candidate CYPs were selected for the characterization of the respective enzymes for their ability to metabolize herbicides. Functional assays of the CYPs have been carried out by assaying the respective recombinant proteins in yeast coexpressing a plant CYP-reductase, either using whole-cell assays *in vivo* or *in vitro* by incubating herbicides with membrane preparations from the respective cultures in the presence of NADPH. As such, this study has had the objective of using a concerted approach to define the CYPs responsible for herbicide selectivity in a globally important cereal crop and a problematic competing wild grass.

## Results

### Treatment with Cloquintocet-Mexyl Safens the Herbicide Pinoxaden in Seedlings of Wheat, but Not Blackgrass

The safener cloquintocet-mexyl is widely used in wheat production to protect the crop from chemical injury caused by a range of postemergence selective herbicides, including pinoxaden, through enhancing their detoxification by CYPs.^3,28^ As cloquintocet-mexyl functions to enhance herbicide tolerance in wheat, its protective effect would not be expected to extend to competing wild grasses. However, this tenet has been challenged in recent years by reports of safeners enhancing herbicide tolerance in ryegrass (*Lolium sp*.).^29^ As safening was to be used in the current study as a fundamental tool to identify detoxifying CYPs of interest, the potential for cloquintocet-mexyl to enhance tolerance toward pinoxaden was performed in wheat and blackgrass. In both cases, the detoxifying activities of the CYPs were determined by monitoring the formation of respective herbicide metabolites, as it proved impossible to measure the enzyme activities directly using plant extracts. For reference, the effect of cloquintocet-mexyl treatment on the toxicity of chlorotoluron was also assessed, as PU herbicides are not used in conjunction with safeners for selective weed control in wheat.

**Figure 2:**
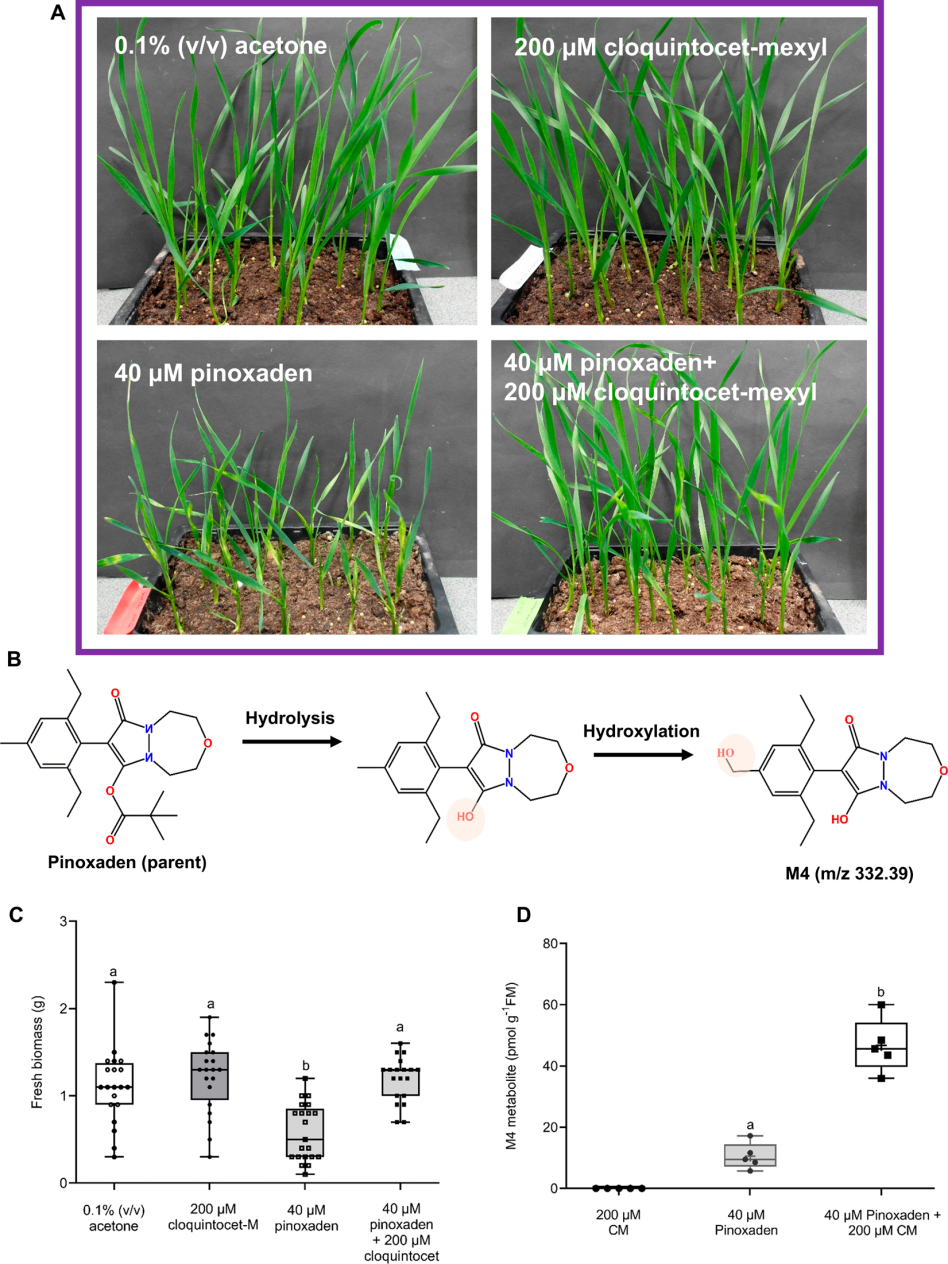
Safening effect of cloquintocet-mexyl toward pinoxaden in wheat seedlings. (A) Seedlings photographed 14 days after treatment with 40 μM pinoxaden, 200 μM cloquintocet-mexyl alone, or following a coapplication of 40 μM pinoxaden and 200 μM cloquintocet-mexyl. (B) The CYP-mediated primary detoxification of pinoxaden in wheat showing the formation of the M4-metabolite (8-(2,6-diethyl-4-hydroxymethyl-phenyl)-9-hydroxy-1,2,4,5-tetrahydro-pyrazolo[1,2]oxadiazepin-7-one). For each treatment, (C) the fresh biomass (FM) of above ground tissue of individual plant was measured, with each box representing the data from 25 individual plants. (D) The effect of coapplication of cloquintocet-mexyl with pinoxaden on metabolite M4 accumulation in the foliage, with each bar derived from 5 biological replicates (*n* = 5). FM and metabolite M4 levels (mean ± SD) of different treatments were compared by one-way ANOVA. The different letters represent significant differences between treatments (Tukey HSD posthoc test; *p* ≤ 0.05).

As determined from a combination of visible assessment, foliar fresh weight, and plant height determinations, treatment of wheat seedlings with cloquintocet-mexyl alone did not inhibit plant growth. Rather, a growth enhancement effect was observed on exposure to the safener as compared with the solvent control treatments (Figure 2A,C). When applied to wheat without cloquintocet-mexyl, pinoxaden caused a reduction of ∼50% in fresh weight and 40% in height compared to controls (Figures 2 and S1A, one-way ANOVA, *p* < 0.05). This inhibitory effect was totally reversed by the coapplication of cloquintocet-mexyl, with comparable results as determined with the control treatments (Figure 2A,C), confirming effective safening. In contrast, the coapplication of the safener with chlorotoluron did not protect winter wheat from herbicide damage (Figure S2).

To determine if the protective effects of cloquintocet-mexyl were linked to enhanced herbicide detoxification in wheat, the metabolism of pinoxaden in the foliage was monitored using UPLC-MS over 24 h ± safener coapplication. Both parent pinoxaden and its primary detoxification product, 8-[2,6-diethyl-4-(hydroxymethyl)phenyl]tetrahydropyrazolo[1,2-*d*][1,4,5]oxadiazepine-7–9-dione (termed metabolite M4),^30^ were determined (Figure 2D). M4 is derived from the aryl hydroxylation of pinoxaden (Figure 2B), a reaction proposed to be catalyzed by CYPs.^30^ Based on the relative disappearance of pinoxaden (Figure S1B; one-way ANOVA, *p* < 0.05) relative to the accumulation of the M4 metabolite (Figure 2D; one-way ANOVA, *p* < 0.05), cotreatment with cloquintocet-mexyl significantly enhanced the hydroxylation of the herbicide. Collectively, these results confirmed that the functional safening of pinoxaden in wheat by cloquintocet-mexyl was associated with the enhanced hydroxylation of the herbicide. The treatments with pinoxaden and safener were then extended to the “Rothamsted” population of blackgrass, which is classically ascribed as being HS, having never been exposed to herbicides.^14,27^ Pinoxaden treatment alone caused severe damage to the blackgrass plants, as determined by all measurement criteria, with no protective effect afforded when the herbicide was coapplied with cloquintocet-mexyl (Figure S3).

### Effect of Cloquintocet-Mexyl Treatment on the Detoxification of Clodinafop-Propargyl in Cell Cultures of Wheat and Blackgrass

The studies with whole plants confirmed the safening of pinoxaden in wheat but not in blackgrass. For the purposes of the comparative transcriptomic and proteomic studies to follow, it was useful to study the effect of the safener on suspension cultures derived from the respective plants, as they represent a mass of relatively homogeneous dividing cells that can be synchronously exposed to chemical treatments in a highly comparative manner.^20,31,32^ While wheat cell lines were already available, in the case of blackgrass, it was first necessary to initiate callus cultures from germinating seeds derived from HS (Rothamsted) and NTSR (Peldon) populations. Through repeated rounds of subculturing, the respective dividing cell lines were then grown as suspension cultures. Prior to running the safening experiment in the cell cultures, the effect of cloquintocet-mexyl on the phytotoxicity of clodinafop-propargyl was first determined in wheat and HS blackgrass seedlings. As determined with pinoxaden, safener treatment reduced the injury invoked by clodinafop in wheat but had no such protective effect in HS blackgrass (Figure S4A). While the safener-linked toxicity studies could not be extended to the respective suspension cultures, it was of interest to determine if cloquintocet-mexyl treatment resulted in the enhanced metabolism of clodinafop-propargyl in the wheat and blackgrass cell lines. Each suspension culture was exposed to 70 μM clodinafop-propargyl ± 100 μM cloquintocet-mexyl, and the cells were analyzed 6 hours after treatment for the presence of herbicide metabolites by UPLC-MS. By reference to available standards and published data,^31^ three metabolites of the parent herbicide were observed, namely clodinafop acid [M + H^+^ = 312.04], formed by the rapid hydrolysis of the propargyl ester; the ester glucoside of clodinafop acid [M + H^+^ = 474.09], and hydroxylated clodinafop [M + H^+^ = 328.038], a primary detoxification product formed by CYP action. In the wheat cultures, the presence of the safener significantly increased the formation of the hydroxylated metabolite as well as the ester glucoside (Figure S4B). In contrast, in both the HS and NTSR blackgrass cultures, the presence of the safener did not increase the formation of either metabolite, though it was notable that the accumulation of these detoxification products was considerably greater in the NTSR blackgrass cells, as compared with the HS cultures (Figure S4B).

### Effect of Cloquintocet-Mexyl Treatment on the Transcriptome of Winter Wheat

**Figure 3:**
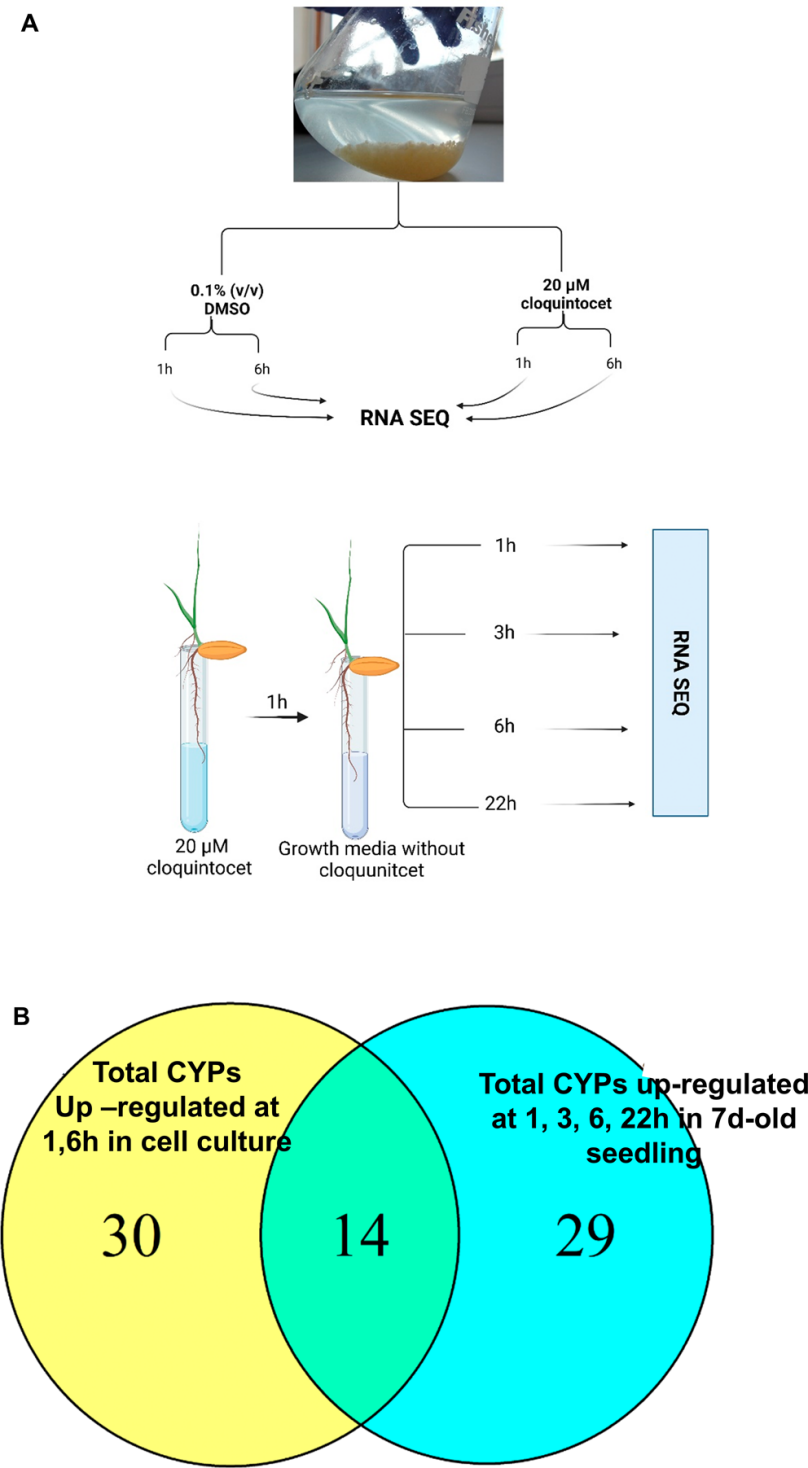
Transcriptome analysis of wheat treated ± the safer cloquintocet-mexyl, showing the experimental design applied to (A) cell cultures and (B) hydroponically grown seedlings. (C) Venn diagram summarizing the induction of wheat CYP family members by cloquintocet-mexyl in the two treatment systems.

Based on the observed enhancement in herbicide metabolism over a 24-h period and preliminary RT-PCR studies with the known safener-inducible gene GSTU6 over 0–6 h, global transcriptome analysis of 7-day-old wheat seedlings treated hydroponically ± cloquintocet-mexyl was performed at 1, 3, 6, and 22 h post-treatment (Figure 3A). Wheat suspension cultures were also utilized as a proxy for plant treatment, with the advantage for a transcriptome study of ensuring synchronous treatment of all plant cells present. With the cultures, the time-course exposure was limited to 1 and 6 h ± cloquintocet-mexyl treatment (Figure 3A)

Using RNA-Seq, a total differential analysis of transcriptional changes was performed between solvent control (0.1% (v/v) DMSO) and safener-treated (i) 7-day-old seedlings and (ii) suspension-cultured cells, respectively, with the number of transcripts up-regulated (log_2_ fold change ≥ 1, FDR ≤ 0.05) or down-regulated (log_2_ fold change ≤ −1, FDR ≤ 0.05) determined at each time point. Based on these selection criteria, in the seedling treatments, a total of around 500 genes were down-regulated by cloquintocet-mexyl and over 1000 were up-regulated, with the greatest perturbation observed 22 h after treatment (Table 1). In the cell cultures, at time points 1 h and 6 h, the numbers of perturbed genes in the wheat suspension cultures were of a similar magnitude to those determined in the equivalent seedling treatments (Table 1), providing confirmation that the cell lines were a useful proxy system to study the complexity of safening at the transcriptional level. The respective differentially expressed unigenes were then identified by sequence homology in both the seedlings (Table S2) and cell cultures (Table S3). Genes linked to xenobiotic detoxification were well represented among the inducible transcripts in both the safener-treated seedlings and suspension cultures, illustrating that safening in wheat extends to all phases of xenobiotic metabolism (Table 1).

**Table 1:** Summary of Classes of Genes Linked to Herbicide Detoxification Induced by Cloquintocet-Mexyl Treatment in 7-Day-Old Wheat Seedlings and Cell Cultures (Values Shown in Parentheses) as Determined at Timed Intervals

	Time after treatment (h)			
Changes in Transcript abundance	1	3	6	22
Total up-regulated	545 (744)	1062	1220 (1056)	1250
Total down-regulated	22 (115)	222	224 (297)	561
Induced CYP450s	25 (34)	40	36 (38)	41
Induced GSTs	56 (50)	77	84 (58)	82
Induced UGTs	89 (57)	105	103 (66)	103
Induced ABC transporters	19 (19)	38	37 (21)	31

**Figure 4:**
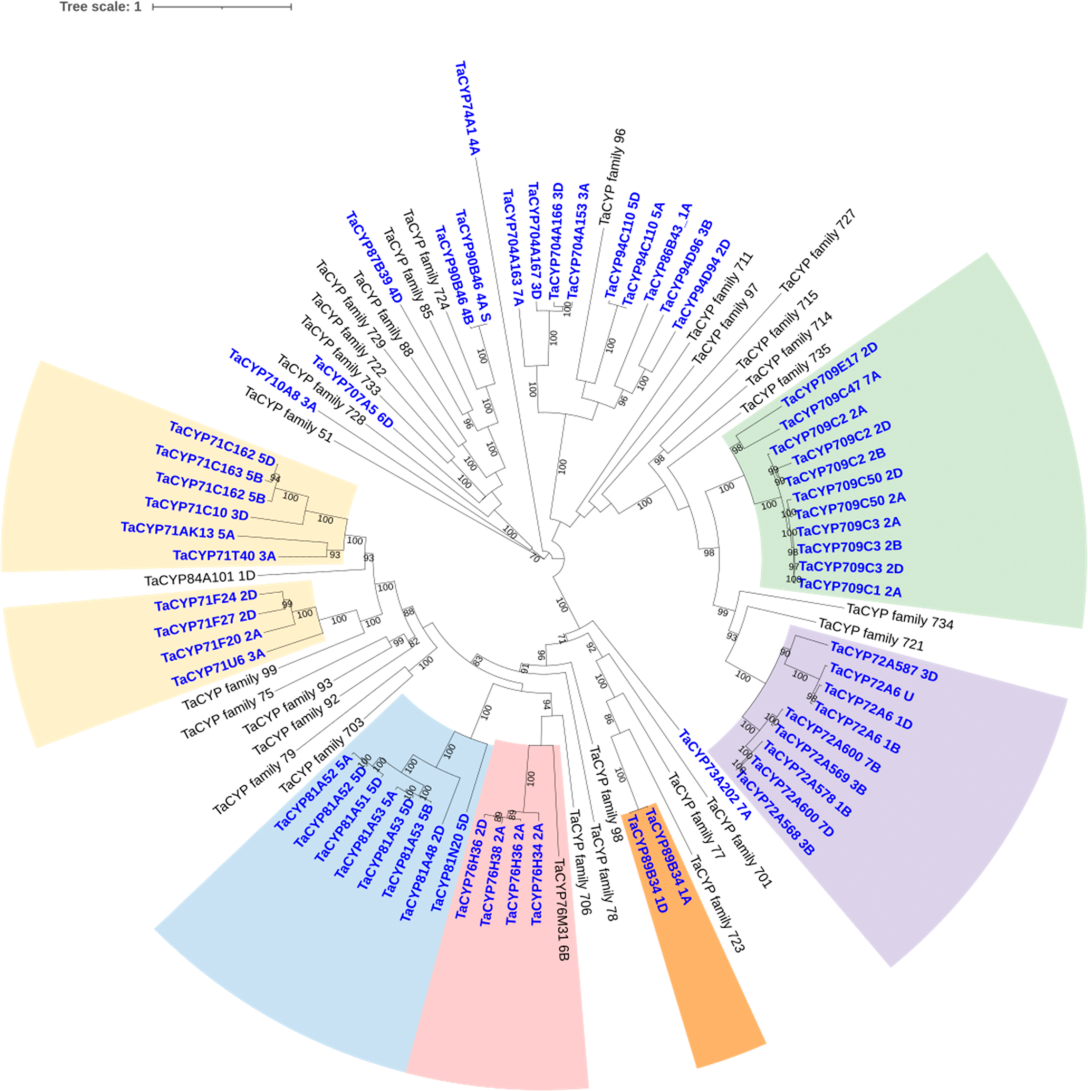
Phylogenetic analysis of CYP families from wheat (*Triticum aestivum*). Numbers on branches represent bootstrap support values >70%, with the scale bar indicating the inferred number of substitutions per site. Sequences in blue correspond to transcripts from wheat up-regulated by the safener cloquintocet-mexyl, with the families selected for subsequent functional characterization shaded in different colors. For those families that were not responsive to cloquintocet-mexyl treatment, only one branch is shown.

With respect to CYPs, over 60 distinct unigenes were detected from a combination of the seedling and cell culture safening RNA-seq studies. Each of the safener-induced CYPs was then identified on the basis of sequence homology to members of the respective wheat gene superfamily compiled from the “Chinese Spring” reference genome^33^ and annotated using standard CYP nomenclature.^34^ A summary of the identities of the CYPs identified is given in Table S4, with the respective family members arrayed on a phylogenetic tree (Figure 4). In total, 14 CYPs were induced in both cell culture and seedling experiments by safener treatment (Figure 3B and Table S4). In view of the consistency in their safener responsiveness, these 14 CYPs were prioritized for further study. In addition, 12 other *Ta*CYPs, based on their relatedness to family members known to be active in herbicide metabolism in other plants or closely associated with functional safening in other cereals (see Supporting Information), were also selected for characterization.

**Figure 5:**
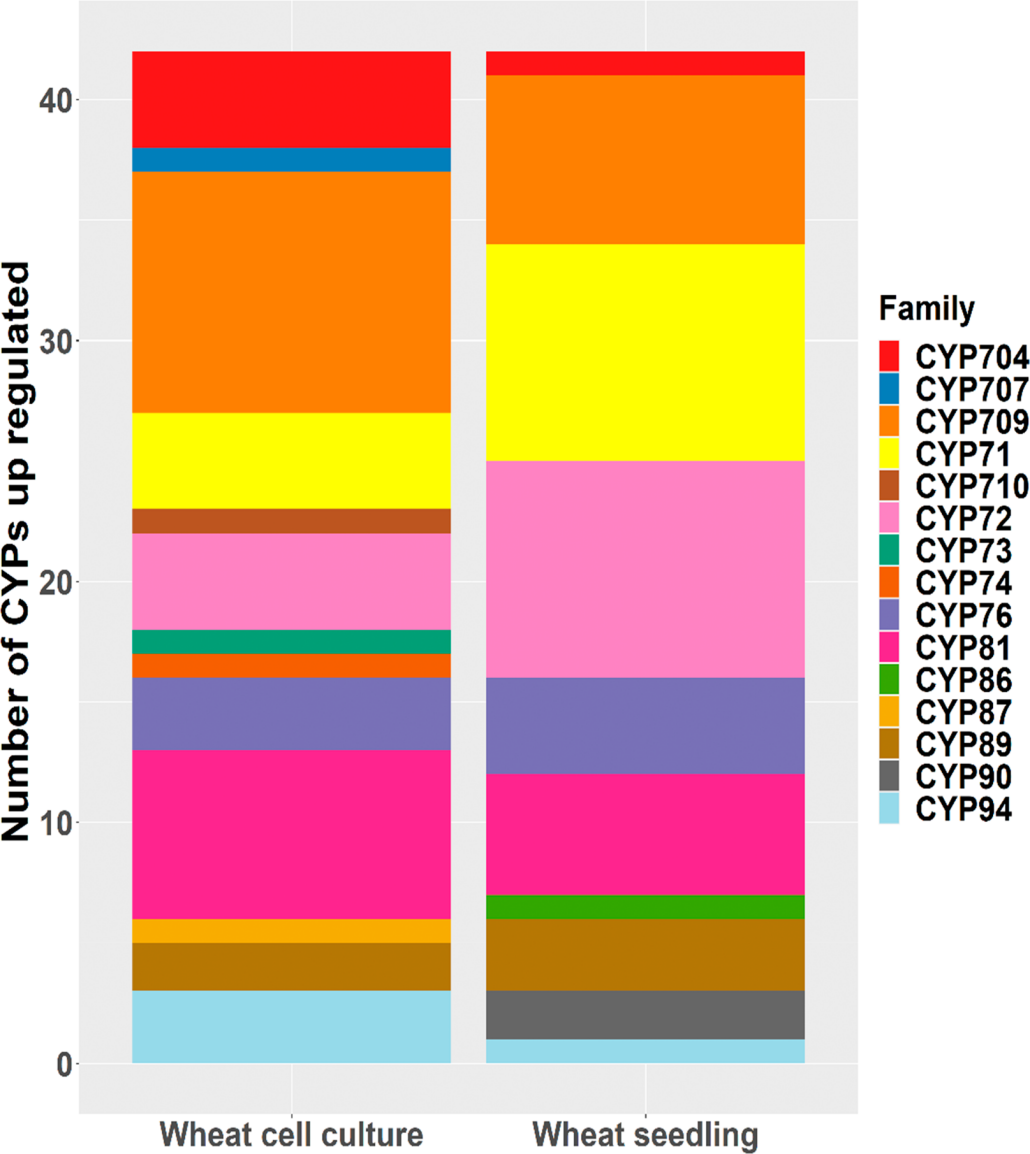
Histograms showing the different CYP families with significantly increased transcript abundance in wheat following treatment with cloquintocet-mexyl in hydroponically grown seedlings and cell cultures, as determined in RNA-seq experiments.

**Figure 6:**
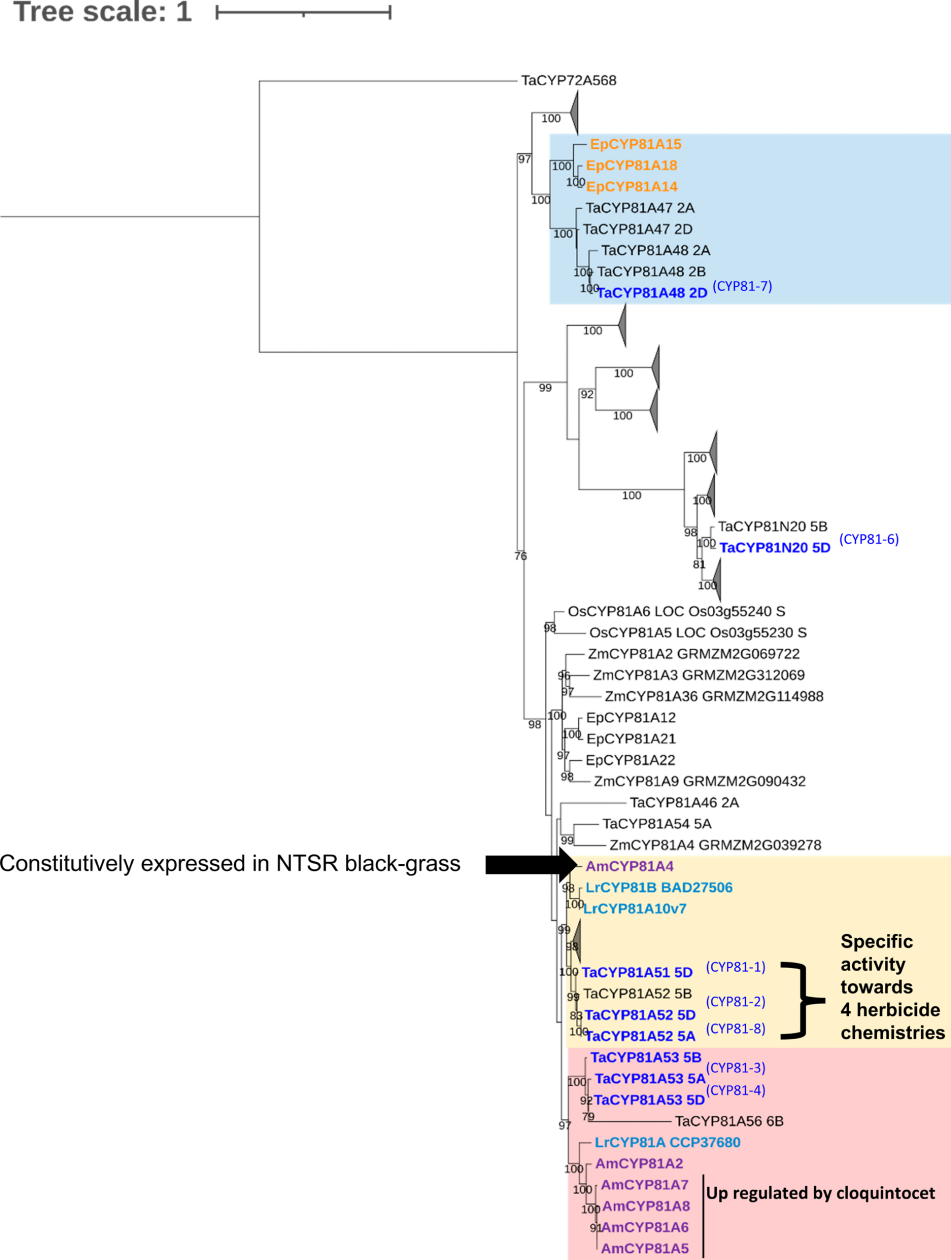
Phylogenetic analysis of CYP81As identified in wheat and blackgrass referenced to family members linked to herbicide metabolism in other wild grasses. Amino acid sequences of wheat (*Ta*, *Triticum aestivum*), maize (*Zm*, *Zea mays*), rice (*Os*,*Oryza sativa*), blackgrass (*Am*, *Alopecurus myosuroides*), late watergrass (*Ep*, *Echinochloa phyllopogon*), and Annual ryegrass (*Lr*, *Lolium rigidum*) were used for maximum likelihood alignment. Wheat CYPs (TaCYPs) induced by treatment with cloquintocet-mexyl are shown in blue text, with sequences related to late watergrass (*Ep*CYP81A14, *Ep*CYP81A15, and *Ep*CYP81A18) whose expression is enhanced by the herbicide bispyribac sodium^37^ shown on a blue background. Sequences related to CYPs up-regulated in resistant blackgrass^22,38^ and annual ryegrass^39^ up-regulated in resistance biotopes are shown in yellow background, and CYPs up-regulated by cloquintocet in blackgrass are shown in a pink background.

In total, 26 *Ta*CYPs were identified for functional expression as the respective recombinant enzymes, representing members of the CYP71, CYP72, CYP76, CYP81, CYP709, and CYP89 families. While the relative safener-responsiveness of each *Ta*CYP was originally defined by RNA-seq analysis (Figures 5, 6, and S5–S7 and Table 2), it was then of interest to accurately quantify the respective abundance of each *Ta*CYP transcript following each treatment using quantitative real-time PCR to obtain an absolute quantification of the respective copy number per 100 ng of cDNA (Table 2). Gene-specific amplification products were obtained in all cases except for *TaCYP81-8*, *TaCYP709-8, TaCYP709-12, TaCYP709-15*, and *TaCP94-1,* where unique primer sequences could not be designed. qRT-PCR analysis confirmed that the majority of the *Ta*CYPs identified as being induced by RNA-seq analysis following safener treatment were present in increased abundance as the respective transcripts, although the scale of the enhancement was generally more modest. The study further confirmed the marked safener-responsiveness of members of the *TaCYP81*, *TaCYP72,* and *TaCYP709* gene families. On analyzing the levels of CYP transcript levels in the foliage of the wheat seedlings following a 24-h safener treatment, of the 21 assayable genes, six CYPs showed copy numbers of >200, which was an order of magnitude greater than that determined for the remainder (Table 2). This analysis also revealed that these six genes were also well represented in untreated samples, suggesting that they were strongly expressed constitutively as well as in response to safener treatment.

**Table 2:** Changes in the Expression of the 26 TaCyps Genes Selected for Detailed Study Following the Exposure of Wheat Seedlings and Cell Cultures to Cloquintocet-Mexyl Over Time^a^^b^

	RNA-seq analysis						qPCR analysis			
	DEG in cells (Log2FC)		DEG in seedlings (Log2FC)				Fold change in transcript abundance in seedlings			
*Ta***CYP**	1 h	6 h	1 h	3 h	6 h	22 h	1 h	3 h	6 h	22 h
CYP81-1	√	√	√√√	√√√√	√√√	√√√√	√√√	√√√√	√√	√√√√
CYP81-2	√√	√	√√√	√√√√	√√√√	√√√√	√√	√√√	√	√√√
CYP81-3	√	√	√√	√	√	√	√√	√√	√	√
CYP81-4	√	√	√√	√	√	√	√√	√	√	√√
CYP81-6		√					**x**	**x**	**x**	**x**
CYP81-7		√					**x**	**x**	**x**	**x**
CYP81-8	√√	√√					Gene specific primers unavailable			
CYP71-15			√	√			**x**	**x**	**x**	√
CYP71-11			√	√	√	√	**x**	**x**	**x**	√
CYP71-17				√√√√	√√√	√√√√	**x**	**x**	**x**	√
CYP709-4			√√√√	√√√√	√√√√	√√√√	**x**	**x**	**x**	√√
CYP709-5	√√	√	√	√√√√	√√√√	√√√√	√	√√√	√√√√	√√√√
CYP709-6	√√	√√		√	√	√	√	√√	√	√√
CYP709-7	√√√	√√	√	√√√	√√√	√√√	**x**	√√√√	√√√	√√√√
CYP709-8				√√√√	√√√	√√√√	Gene specific primers unavailable			
CYP709-15	√√	√√					Gene specific primers unavailable			
CYP709-12	√√	√					Gene specific primers unavailable			
CYP72-1	√	√	√	√√√	√√√	√√√√	√	√√√	√√√	√√√√
CYP72-2			√	√√√√	√√√√	√√√√	**x**	√√	√√√	√√√√
CYP72-4	√	√	√	√√	√√	√√	√	√√√	√√	√√
CYP72-6	√	√	√√	√√√	√√	√√√	√	√√√	√√√	√√√
CYP72-7				√		√	**x**	√√	√	√
CYP76-3		√	√	√	√√	√√√	**x**	**x**	√	√√√
CYP89-1	√	√	√	√	√	√	√	√√	√	√√
CYP89-2	√	√	√√	√√	√	√	**x**	√√	√	√
CYP94-1				√		√	Gene specific primers unavailable			

a The quantification of the respective differentially expressed genes (DEGs) was compared as determined by both RNA-Seq data and quantitative PCR.

b √ = N fold between 2 and 5, √√ = N fold between 5 and 10, √√√ = N fold between 10 and 20, √√√√ = N fold > 20

### Changes in CYP450 Expression in Safener-Treated Seedlings and Suspension Cultures of Wheat as Determined by Proteomics

To determine changes in the expression of *Ta*CYP polypeptides *in planta*, the signature peptides from the respective coding sequences present in microsome preparations isolated from wheat seedlings or suspension cultures treated ± cloquintocet-mexyl were quantified by proteomics (Figure S8A,B). As the expression of the respective transcripts peaked between 6 and 24 h after exposure to the safener (Table 1), changes in the relative abundance of the encoded proteins were determined at 24 h to allow for the delay in *de novo* protein synthesis. After preparing microsomes, membrane-associated proteins were first resolved by SDS-PAGE (Figure S8C,D), with stained protein bands of interest treated with trypsin, and the peptide digests were then analyzed by HPLC-MS-based quantitative proteomics. Changes in the relative abundance of polypeptides following safener treatment were then analyzed using volcano plots (Figure S8E,F), and the respective proteins showing significant perturbation (as determined by Student’s *t*-tests) identified for both the wheat seedling (Table 3A) and cell culture studies (Table 3B). Cloquintocet-mexyl treatment of wheat seedlings and cell cultures resulted in the perturbation in abundance of 221 and 82 membrane-associated polypeptides, respectively; of these proteins, 131 and 72, respectively, were enhanced by safening. With respect to changes in CYP polypeptide abundance, safener treatment of the seedlings significantly enhanced a total of 14 CYPs comprising members of the *Ta*CYP72, *Ta*CYP81, *Ta*CYP704, *Ta*CYP709 and *Ta*CYP89 families (Table 3A). In the cell cultures, CYP protein enhancement by cloquintocet-mexyl was less marked, although seven of the nine identified polypeptides shared a common identity to the *Ta*CYPs induced in the seedling studies (Table 3B).

**Table 3:** Changes in the Abundance of CYPs Identified in Microsomes Prepared from A. Wheat Seedlings and B. Cell Cultures Following Exposure to Cloquintocet-Mexyl as Defined by Fold Differences in Their Quantification by Proteomics Relative to Those of Untreated Controls

UniPro ID	BlastP annotation	Magnitude of change cloquintocet vs DMSO	Similar sequences from RNA SEQ	Confirmed transcript expression by qRT-PCR
A				
**A0A3B5Z3K0**	TaCYP72A15-like	**1.62**	TaCYP72-2	YES
**A0A3B5Y4M3**	TaCYP72A15-like	**2.16**	TaCYP72-1	YES
**A0A3B6TK18**	TaCYP72A15-like	**2.26**	TaCYP72-4	YES
**A0A3B6SLB0**	TaCYP72A15-like	**4.32**	TaCYP72-6	YES
**Q2 V066**	TaCYP72A14-like	2.49	NO	No
**A0A3B6MY76**	TaCYP81Q32-like	**2.66**	TaCYP81-4	YES
**A0A3B6LTK6**	TaCYP81Q32-like	**2.75**	TaCYP81-5	NO
**A0A3B6LTM1**	TaCYP81Q32-like	3.33	NO	NO
**A0A3B6MXY8**	TaCYP81Q32-like	**4.3**	TaCYP81-1	YES
**A0A3B5YXS8**	TaCYP89A2-like	**3.34**	TaCYP89-1	YES
**A0A3B6ATB1**	TaCYP709B1-like	3.24	TaCYP709-14	NO
**A0A3B6AV58**	TaCYP709B2-like	**1.89**	TaCYP709-6	NO
**A0A3B6EB45**	TaCYP704C1-like	**3.54**	**YES**	NO
**A0A077RQA6**	TaCYP704C1-like	5.86	NO	NO
B				
**A0A3B5Y4M3**	TaCYP72A15-like	0.60	TaCYP72-1	YES
**A0A3B6LTK6**	TaCYP81Q32-like	0.58	TaCYP81-5	YES
**A0A3B6MXY8**	TaCYP81Q32-like	**0.91**	TaCYP81-1	YES
**A0A3B6MYN4**	TaCYP81Q32-like	0.97	No	NO
**A0A3B5YXS8**	TaCYP89A2-like	**0.44**	TaCYP89-1	YES
**A0A3B5XZ58**	TaCYP89A2-like	**0.63**	TaCYP89-2	YES
**A0A3B6EB45**	TaCYP704C1-like	**2.45**	**YES**	NO
**A0A3B6ATB1**	TaCYP709B1-like	1.38	TaCYP709-14	NO
**A0A3B6AV58**	TaCYP709B2-like	**1.41**	TaCYP709-6	NO

On comparison with the transcriptome data, while 10 families of *TaCYP* genes were significantly induced by cloquintocet-mexyl in seedlings (Table 2 and Figure 5), only 5 of these were found to be enhanced at the protein level (Table 3A). Thus, while the transcription of genes encoding *Ta*CYP71s and *Ta*CYP76s was markedly increased by safener treatment, there was no detectable enhancement in the abundance of the respective proteins. Where *Ta*CYP genes and proteins were both enhanced, the magnitude of transcript induction (Table 2) greatly exceeded the observed increase in the abundance of the encoded polypeptides (Table 3). However, in the case of the *Ta*CYPs families 81 and 72, comparative analysis did indicate a linear correlation between transcript and protein induction following exposure to cloquintocet-mexyl. Collectively, these results revealed a complex regulation of CYP expression by cloquintocet-mexyl in wheat, exerted at both the transcriptional and post-transcriptional levels to control the expression of the respective enzymes. Based on the correlation of their enhancement by this combination of pre- and post-transcriptional regulation mechanisms, the results highlighted the apparent importance of families *Ta*CYP72 and *Ta*CYP81 in the safening response in wheat.

### Activity of Wheat CYPs Toward Herbicides When Expressed as Recombinant Enzymes in Yeast

The selected 26 *Ta*CYPs were expressed as the respective recombinant (rCYP) enzymes in *Saccharomyces cerevisiae* cultures which had been cotransformed with a plant (*Arabidopsis thaliana*) CYP-reductase to optimize catalytic activity.^35^ Each *Ta*CYP sequence was optimized for yeast expression as the respective STRP-tagged recombinant polypeptide, and following culturing and induction, cell extracts were used to prepare microsomes for testing in *in vitro* assays, utilizing NADPH as the reducing cofactor.^20^ The use of the STRP-tag enabled the routine monitoring of recombinant protein expression using anti-STRP serum by immunoassay, as well as serving as a reference peptide sequence for direct rCYP quantification using an internal standardized proteomics protocol, thereby allowing for accurate enzyme-specific activities to be determined.^20^ The levels of picomoles of rCYP determined per milligram of total microsomal protein varied significantly for each of the 26 recombinant enzymes tested (Table S5).

In the case of *Ta*CYP709–4, *Ta*CYP709–5, *Ta*CYP709–6, *Ta*CYP709–7, *Ta*CYP709–8, and *Ta*CYP94–1, the levels of expression of the rCYPs were below the limit of detection by proteomics (Table S5). Similarly, when tested by western immunoblotting using the anti-STRP serum, no fusion proteins could be detected in the respective microsomes (data not shown), confirming the very low expression of these constructs in recombinant yeast. As such, further analysis of the enzyme activities of these six *Ta*CYPs was not performed. Screening for CYP activity using the microsome preparations focused on herbicides widely used for selective weed control in wheat, with the DIMs cycloxydim and tralkoxydim, the FOPs diclofop acid and clodinafop acid, the PU chlorotoluron, the triazolopyrimidine pyroxsulam, the pyrazoles pinoxaden and pyrasulfotole, and the SUs nicosulfuron and mesosulfuron being selected as representative substrates. Following their incubation with the recombinant yeast microsomal preparations using assay conditions optimized for maize CYPs,^20^ the presence of CYP-derived biotransformation products was then determined by UPLC-MS, using reference or published metabolite MS spectra for identification and quantification. In each case, it was then possible to calculate the specific activity of each CYP based on the quantification of the respective recombinant enzyme by quantitative proteomics. Primary screens for enzyme activity toward these herbicides using the microsomes were unable to detect CYP-based biotransformation activity with cycloxydim, pinoxaden, pyrasulfotole, mesosulfuron, diclofop acid, or clodinafop acid with any of the expressed 20 recombinant CYPs. However, activities toward pendimethalin, chlorotoluron, pyroxsulam, and tralkoxydim were readily determined, and these encompassed a range of demethylation and hydroxylation reactions (Figure 1). In the case of chlorotoluron, both *N*-demethylation and ring hydroxylation of the herbicide could be determined. While all of the enzymes tested showed activity toward at least one herbicide chemistry (Table 4), only three *Ta*CYPs, all belonging to family 81, were active toward all four herbicides, namely the closely related *Ta*CYP81-1 and *Ta*CYP81-2, as well as *Ta*CYP81-7 and *Ta*CYP81-8. The sequences of these *Ta*CYP81s clustered with three *Ec*CYP81s from *Echinochola phyllopogon* (Figure 6), which are also known to detoxify herbicides.^36^

**Table 4:** Activities of Recombinant Wheat CYPs Following Expression of the Respective Recombinant Enzymes in Yeast as Assayed as the Respective Microsomal Preparations and Determined with a Herbicide Substrate Concentration of 200μM^a^

	Specific activity (pkat mg^–1^ recombinant CYP)					
*Ta*CYPs	Pyroxsulam hydroxy	Pendimethalin demeth	Chlorotoluron demeth	Chlorotoluron hydroxy	Tralkoxydim hydroxy	Mesosulfuron demeth
**Family 81**						
CYP81-1	309 ± 148	12,655 ± 747	12,960 ± 2308	4058 ± 592	18,076 ± 1771	ND
CYP81--2	1487 ± 798	18,494 ± 5350	33,328 ± 8911	9228 ± 1576	90,997 ± 2026	ND
CYP81-3	ND	ND	ND	ND	576 ± 7.8	ND
CYP81-4	ND	450 ± 378	ND	ND	ND	ND
CYP81-6	ND	67 ± 50	ND	ND	ND	ND
CYP81-7	ND	388 ± 59.3	664 ± 47	568 ± 198	30 ± 0.5	ND
CYP81-8	463 ± 103	22,597 ± 4854	3818 ± 169	7722 ± 1138	7697 ± 223	ND
**Family 71**						
CYP71-15	ND	ND	38 ± 98	644 ± 147	ND	ND
CYP71-11	ND	91,978 ± 8707	5193 ± 151	34,103 ± 888	216 ± 3	ND
CYP71-17	ND	ND	ND	ND	ND	ND
**Family 76**						
CYP76-3	ND	2856 ± 468	868 ± 31	194,284 ± 3197	ND	ND
**Family 89**						
CYP89-1	ND	104 ± 152	61 ± 50	148 ± 13	ND	ND
CYP89-2	ND	791 ± 1083	ND	ND	ND	ND
**Family 709**						
CYP709-15	ND	ND	ND	1015 ± 174	ND	ND
CYP709-12	ND	ND	ND	ND	ND	ND
**Family 72**						
CYP72-1	ND	176 ± 270	ND	327 ± 208	ND	ND
CYP72-2	ND	42 ± 35	ND	319 ± 299	9261 ± 617	ND
CYP72-4	ND	17 ± 10	ND	ND	748 ± 33	ND
CYP72-6	ND	242 ± 164	ND	353 ± 37	463 ± 9	ND
CYP72-7	ND	ND	ND	ND	ND	ND

a The specific activities represented mean ± SD (*N* = 3) for each herbicide. ND = no activity detected.

Uniquely, of all the enzymes assayed, only the CYP81s were active toward pyroxulam. In terms of specific activity, the other highly active herbicide-detoxifying CYPs were found in family 71 (*Ta*CYP71-11) and family 76 (*Ta*CYP76-3), which respectively showed the greatest detoxifying demethylating activity toward pendimethalin and hydroxylating activity with chlorotoluron of any of the enzymes tested (Table 4). Of the remaining *Ta*CYPs tested, activity toward herbicides was either an order of magnitude lower or below the level of detection, being typically limited to the demethylation of pendimethalin or the hydroxylation of chlorotoluron.

### Assay for CYP Activity Toward Clodinafop and Pinoxaden Using Recombinant Yeast Cells

While the use of the microsomal recombinant CYPs had proven useful in identifying candidate enzyme responsible for acting on pendimethalin, tralkoxydim, chlortoluron, and pyroxulam, it was surprising that none of the enzymes assayed showed activity toward pinoxaden or the FOP herbicides diclofop or clodinafop. In the current study, both pinoxaden and clodinafop have been shown to undergo CYP-catalyzed metabolism in wheat plants and cell cultures following safener treatment (Figures 2, S1 and S4). The absence of activity suggested that either none of the assayed enzymes were active toward these classic CYP substrates, that the respective activities were very low, or that the assay conditions were not suitable for their detection. Additionally, the lack of activity using the recombinant yeast system might also be due to poor coupling between the cereal CYP and the heterologous CPR derived from *Arabidopsis*. As an alternative to the *in vitro* assay of the enzymes expressed in microsomal preparations, herbicides were added to the media of the respective CYP-recombinant yeast cell cultures, and any metabolites formed were then detected in the respective cultures *in vivo*. While such a procedure cannot be used to define enzyme-specific activity, it has the advantage of maximizing the potential for any biotransformation by the recombinant CYP by extending the period of exposure to the herbicides.^20^ To ensure that the recombinant plant CYPs were indeed able to metabolize herbicides, two CYP81s from *Echinochola phyllopogon, Ec*CYP81-12 and *Ec*CYP81-21, which have been reported to show activities toward pinoxaden and diclofop, respectively, when expressed in microbial hosts,^36^ were included in parallel screens of the yeast cultures for reference. In each case, the recombinant cultures were incubated for 24 h with pinoxaden, clodinafop, tralkoxydim, or chlortoluron, the latter two herbicides being included as positive controls. In each case, the media were then analyzed for the presence of CYP-derived oxidation products.^20^ Using this procedure, yeast cultures expressing *Ta*CYP81-1 and *Ta*CYP81-48 were shown to accumulate metabolites of both clodinafop and pinoxaden.

In the case of clodinafop, the primary hydroxylation product [M + H^+^ = 328.03] could be detected. This metabolite had similarly been identified in the metabolism studies of wheat cell cultures with this herbicide (Figure S4). The results with pinoxaden were more surprising. While *in planta* studies with wheat seedlings had shown that the primary oxidation product of pinoxaden was the metabolite M4 (Figure 2D), in the studies with the recombinant *Ta*CYPs expressed in yeast, the major product was the carboxylate derivative (Table 5). Comparison with the yeast cultures transformed with the *Ec*CYP81s from *E. phyllopogon* showed that, while *Ec*CYP81-12 was inactive toward both substrates, *Ec*CYP81-21 was able to form the carboxylate of pinoxaden but showed no activity toward clodinafop under the assay conditions used. To confirm their status as functional enzymes in the recombinant yeast host, both *Ta*CYP81-1 and *Ta*CYP81-48 and the two *Ec*CYP81s were shown to be active toward tralkoxydim and chlorotoluron (Table 5), confirming that all four proteins were catalytically viable.

**Table 5:** Activities of Recombinant Wheat CYPs Toward Pinoxaden and Clodinafop as Assayed in the Respective Yeast Cultures Incubated for 24 h with the Respective Herbicide^a^

	Level of metabolite (peak area unit mL^–1^ cell culture)					
CYP81	OH-tralkoxydim	OH-pinoxaden	COOH-pinoxaden	OH-clodinafop	Demethylated chlorotoluron	OH-chlorotoluron
*Ep*CYP81–12	387750 ± 17088	ND	ND	ND	1862 ± 545	1114 ± 247
*Ep*CYP81–21	357786 ± 12732	ND	1464 ± 24	ND	1528 ± 293	1364 ± 59
*Ta*CYP81–1	205428 ± 10058	ND	380 ± 152	304 ± 104	19954 ± 1730	1768 ± 172
*Ta*CYP81–7	1164 ± 822	ND	640 ± 88	266 ± 34	4480 ± 174	1076 ± 292

a The level of metabolite is expressed as mean ± SD, *N* = 3. ND – was not detected.

### Identification of CYPs Linked to Herbicide Detoxification in Blackgrass

Differential transcript expression was again used as a strategy for identifying key CYPs involved in herbicide detoxification in blackgrass. Thus, while herbicide safener treatment did not enhance CYP-mediated detoxification of herbicides in HS blackgrass, numerous NTSR populations of this weed showed enhanced CYP-based metabolism toward multiple herbicides.^22,38^ It therefore followed that, as compared with the transcriptome of untreated HS plants, those CYPs that were upregulated by NTSR were potential candidates for detoxifying herbicides, while those induced by safener were less likely to possess such activity. Previous transcriptome studies using seedlings had identified multiple herbicide-detoxifying genes associated with NTSR, including those in resistant populations directly evolved from the reference HS plants used in the current study.^22^ Among these NTSR-associated genes were several CYPs, notably members of the CYP71, CYP72, and CYP81 families (Table S6). A differential transcriptome study was then conducted using the HS blackgrass cultures treated with the safener cloquintocet-mexyl under conditions identical to those used with the wheat cells. While safener treatment of HS blackgrass cells had not been shown to enhance the detoxification of either pinoxaden or clodinafop in the feeding studies with the respective suspension culture (Figures S3 and S4), exposure to the safener caused the enhanced expression of 82 different genes linked to xenobiotic detoxification, including 37 *Am*CYPs (Table 6).

**Table 6:** CYP450s Induced by Cloquintocet-Mexyl in Herbicide-Sensitive Blackgrass

CYP family	
81	*AmCYP81A5, AmCYP81A6, AmCYP81A7,*and *AmCYP81A8*
71	*AmCYP71C5* and *AmCYP71C3*
72	*AmCYP72A6, AmCYP72A7, AmCYP72A8,* and *AmCYP72A9*
709	*AmCYP709C1, AmCYP709C2, AmCYP709C3, AmCYP709C4, AmCYP709C5, AmCYP709C6, AmCYP709C7, AmCYP709C8, AmCYP709C9, AmCYP709C10,* and *AmCYP709H1*
89	*AmCYP89B1, AmCYP89B2, AmCYP89B3, AmCYP89B4, AmCYP89B5, AmCYP89B6, AmCYP89B7, AmCYP89B8, AmCYP89B9, AmCYP89B10, AmCYP89B11, AmCYP89B12, AmCYP89B13, AmCYP89B14, AmCYP89B15,* and *AmCYP89B16*

**Figure 7:**
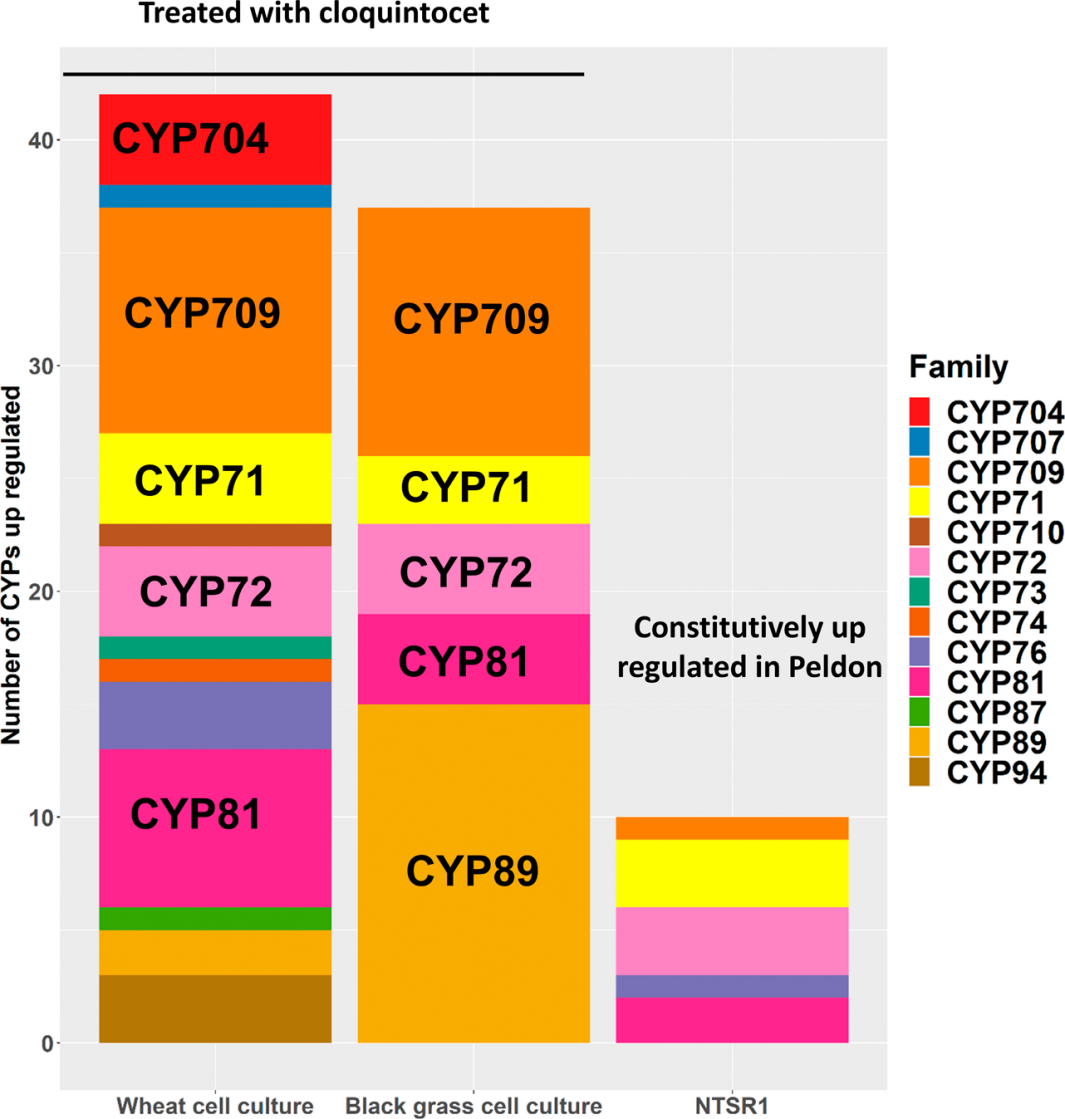
Comparison of CYP families induced in herbicide sensitive (HS) blackgrass and wheat cell culture after 24 h of treatment with cloquintocet-mexyl. For reference, the CYPs that are constitutively upregulated in NTSR vs HS blackgrass plants are also shown.

In comparison with the wheat cultures, a less diverse range of CYPs was induced by cloquintocet-mexyl in blackgrass cells, being dominated by members of the CYP89 and CYP709 families. In contrast, relatively fewer gene members of those CYP families linked to herbicide detoxification in wheat (namely CYP71, CYP72, and CYP81) were enhanced (Figure 7). With respect to the *Am*CYPs linked to NTSR, each optimized coding sequence was expressed in yeast, and the recombinant microsomal preparations were assayed for activity toward herbicides (Table 7). Of the 13 *Am*CYPs tested, eight either failed to express measurable levels of the respective enzyme or showed no activity toward the herbicides tested. Modest activity toward a restricted number of herbicides was observed with a single representative of each of the CYP71, CYP72, CYP76, and CYP704 enzymes tested (Table 7). However, the most active enzyme toward multiple herbicides was *Am*CYP81A4, an enzyme whose respective transcripts were strongly enhanced in NTSR blackgrass but not by safener treatment. The association of herbicide-detoxifying activity with *Am*CYP81A4 was all the more intriguing, as the related enzymes, *AmCYP81A5*, *AmCYP81A6*, *AmCYP81A7*, and *AmCYP81A8*, which were not enhanced in NTSR blackgrass but were induced by safener treatment, showed no activity toward the herbicides.

**Table 7:** Activity of CYPs from Blackgrass Toward Herbicides with Their Respective Perturbation by Herbicide Safener Treatment, or by the Evolution of Non-Target Site Herbicide Resistance Shown^a^

	Specific activity (pkat mg^–1^ recombinant CYP)					
CYPs	OH-pyroxsulam	Demethylated pendimethalin	Demethylated chlorotoluron	OH- chlorotoluron	OH- tralkoxydim	Demethylated mesosulfuron
Family 81						
CYP81A4	ND	14,917 ± 596	55,061 ± 6472	14,613 ± 323	335 ± 257	ND
CYP81A2	ND	ND	ND	ND	ND	ND
Family 72						
CYP72A5	ND	ND	ND	ND	ND	ND
CYP72A4	NT	NT	NT	NT	NT	NT
CYP72A3	NT	NT	NT	NT	NT	NT
CYP709C1	NT	NT	NT	NT	NT	NT
Family 71						
CYP71 × 1	ND	ND	168.66 ± 27.5	130.37 ± 36.12	334.72 ± 256.74	ND
CYP71C3	NT	NT	NT	NT	NT	NT
CYP71 × 2	NT	NT	NT	NT	NT	NT
Family 76						
CYP76H1	NT	NT	NT	NT	NT	NT

a Mean ± SD specific activities (*N* = 3) are shown for each herbicide.

## Discussion

Our studies have revealed that, despite the large size of the CYP superfamily in wheat (1285 genes),^33^ only a small number are associated with herbicide metabolism, notably members of the CYP71, CYP72, CYP76, and CYP81 families. In all cases, the CYPs from these families were enhanced at the levels of both transcript and protein following treatment with the safener cloquintocet-mexyl. While an extensive selection of herbicides used for selective weed control in wheat was tested as substrates in the microsomal assays, activity could only be detected toward the herbicides pyroxsulam, tralkoxydim, chlorotoluron, and pendimethalin. The lack of activity in the wheat recombinant CYP microsome assays toward classic FOP chemistries (diclofop, clodinafop) and pinoxaden was surprising, given the results of the respective herbicide metabolism studies *in planta.* Moreover, the status of these compounds as classic postemergence herbicides that owe their selectivity toward wild grasses due to their rapid detoxification in the crop by CYPs induced by coapplication with safener is well established.^3,31^

Previous studies have confirmed that recombinant CYPs from *Echinochola phyllopogon* and other wild grasses have activities toward FOP chemistries, albeit the respective assays involved incubating the herbicides with yeast cultures expressing the enzymes rather than *in vitro* microsomal preparations.^36,40^ Using a similar “whole-cell” feeding approach, it was subsequently demonstrated that *Ta*CYP81-1 and *Ta*CYP81-7 were indeed active toward clodinafop and pinoxaden, highlighting the potential limitations of the quantitative *in vitro* microsomal assays. While pyroxsulam and tralkoxydim are commonly used with the safener cloquintocet-mexyl in wheat to reduce crop injury,^3,18^ the pre-emergence herbicide pendimethalin and the PU chlorotoluron are not applied with safeners, being reliant upon constitutively expressed CYP activities to confer herbicide tolerance. As such, two groupings of key detoxifying activities can be functionally described in wheat. The first are those CYPs with activity toward herbicides, such as pendimethalin or chlorotoluron, which are present constitutively and whose induction by safeners is not critical for selectivity. The second group is the CYPs active toward postemergence herbicides such as tralkoxydim and pyroxsulam, which are dependent upon their induction by cloquintocet-mexyl treatment to confer tolerance. Based on such criteria, the profile of enzyme activities and inducibility by safener would place CYP81-1, CYP81-2, and CYP81-8 as the prime candidates for the safener-dependent detoxification of tralkoxydim and pyroxsulam. In the case of the constitutive CYP activities, 14 of the CYPs showed activity toward pendimethalin, and as such, their cumulative activities could well account for the constitutive metabolism of the herbicide. Four enzymes were highly active toward pendimethalin, namely, the three CYP81s linked to safening, as well as CYP71-11. However, as none of the CYP71s were found to be expressed at measurable levels in wheat treated ± safener, it would seem most likely that the CYP81s were responsible for pendimethalin detoxification *in planta*. By way of comparison, it is noteworthy that in *Arabidopsis thaliana* members of the CYP706 family metabolize pendimethalin by hydroxylation of the side chain.^48^ However, in wheat, no CYP706s could be identified either by RNA-seq or proteomic analysis, suggesting that different classes of CYPs are linked to pendimethalin metabolism in dicots (*Arabidopsis*) and monocots (wheat and blackgrass).^41^ The role of the different CYPs in chlorotoluron metabolism was more subtle, based on the potential for the herbicide to undergo either demethylation or hydroxylation. While hydroxylation achieves the total detoxification of chlorotoluron, the *N*-demethylated product retains phytotoxic activity.^9,10^ As such, only CYPs showing hydroxylating activity toward chlorotoluron are likely to confer tolerance to the herbicide. Intriguingly, eight of the *Ta*CYPs were active toward chlorotoluron, with each catalyzing both reactions, albeit with different preferences. Based on their specific activities, the most highly active enzymes were CYP81-1, CYP81-2, and CYP76-3. While the two CYP81s showed the greatest activity in demethylating chlorotoluron, CYP76-3 was the most active of all enzymes tested in hydroxylating the herbicide and would therefore be a prime candidate for its detoxification. Significantly, members of the CYP76 family are known to detoxify chlorotoluron by hydroxylation in dicotyledonous plants.^42,43^

In terms of the ability to detoxify multiple herbicide chemistries, matched to the relative abundance in expression based on quantitative transcriptomics and proteomics, CYP81-1 appears to have a fundamental role in determining safener dependent herbicide selectivity in wheat. The dependence on a limited number of members of the CYP81 family to metabolize and hence detoxify multiple herbicides is an emerging trend in studies in both crops, such as maize^20^ and rice,^19^ as well as in competing wild grasses, such as water-grass^36,40^ and rye-grass,^44^ as demonstrated with the enzyme CYP81A4 in the current study in blackgrass. It is apparent that a combination of the structure and enzyme chemistry of the CYP81 family provides an adaptable scaffold to oxidatively biotransform a diverse range of xenobiotic chemistries, as has also been observed with single families of CYPs in both insects^45−47^ and humans.^48−51^ The studies reported here in wheat demonstrate that closely related CYP81 individual family members show a wide range of specific activities toward the herbicides tested, with intriguing potential for mutations and simple recombination events to rapidly evolve new catalytic detoxifying activities. This functional evolvability has the potential to provide weeds with a mechanism to rapidly acquire metabolic resistance to new herbicides, in addition to the more commonly observed evolution of NTSR through enhanced expression of the detoxifying CYP. Certainly, a deeper molecular understanding of the enzyme chemistry of these plant CYPs will be instrumental in linking their structure to function and predicting their roles in detoxifying different herbicide chemistries in both crops and weeds, with implications in predicting selectivity traits as well as their propensity to contribute to resistance in the future.^11^

As determined with other classes of detoxifying enzymes,^3,52−54^ herbicide safener treatment of wheat seedlings and cell cultures caused the up-regulation of multiple gene family members through a complex interaction of both transcriptional and post-transcriptional regulation. In the current studies in wheat, multiple CYP genes were shown to be induced, in some cases massively, at the level of increased transcript abundance, whereas the proteomic studies showed a much more restricted range of respective membrane-bound CYPs being enhanced, invariably at an order-of-magnitude level of increased abundance. Notably, with CYP71 and CYP76 family members, a major enhancement in the abundance of the respective transcripts failed to produce measurable levels of detectable microsomal CYP proteins, as determined by proteomics. These results suggest that safener induction of CYPs in wheat is subject to extensive post-transcriptional regulation, with the large number of induced genes being effectively filtered down to a smaller number of enhanced functional proteins. This level of post-transcriptional regulation has also been reported with the induction of mammalian CYPs,^48^ as well as in cereals when linked to safening by GSTs^53,54^ and members of the ABC transporter family.^55^ This unique insight is a consequence of the inclusion of proteomic analysis in the current safening study and points to the limitations of relying on transcriptomics alone to predict the functional basis of safener action.

Safener studies in HS blackgrass cultures were also revealing, identifying a large number of genes being induced, which were potentially linked to herbicide detoxification. Safener-inducible genes in blackgrass included multiple CYPs, including four CYP81s (*AmCYP81A5*, *AmCYP81A6*, *AmCYP81A7*, and *AmCYP81A8)*, which were similar in sequence to family members in other crops and weeds with known herbicide-detoxifying activity. In contrast, the related blackgrass *Am*CYP81A4 did show activity toward herbicides, but this gene was unresponsive to safeners while being strongly enhanced in NTSR cultures. The differential expression of these CYP81s by cloquintocet-mexyl and NTSR, respectively, adds further evidence of the fundamental differences in gene regulation associated with safening vs the evolution of metabolism-based herbicide resistance in blackgrass.^56^ However, the safener-inducibility of CYP81 members in this problematic weed and the potential for such responsive CYPs to undergo mutations or recombination to afford herbicide-detoxifying activities is a potential concern, given the genetic plasticity of this weed species.^27^ In fact, the overall results of our studies show that the basis of CYP-mediated herbicide selectivity in wheat hinges on a very small number of key family members in both the crop and this competing wild grass. While such a finding is encouraging in terms of the potential to apply principles of enzyme screening and engineering to both predict and, in the future, manipulate herbicide detoxification in wheat, it does point to the potential for future problems in weed control through our over-reliance on a small number of agronomically important genes conferring herbicide selectivity. Certainly, future strategies to incorporate the use of safeners in the selective control of wild grasses, which are prone to evolve nontarget site resistance, should incorporate rotations in chemistry that can circumvent overdependence on inducible CYP-based detoxification systems and instead exploit other mechanisms of differential herbicide metabolism, such as glutathione transferases^16^ or ABC transporters.^55^

## Materials and Methods

### Plant Material

Seeds of winter wheat (*Triticum aestivum*) variety Graham were obtained from Syngenta, Bracknell, UK. Seeds of NTSR and HS blackgrass with defined herbicide tolerance traits were available from previous studies.^38,55^ Wheat seeds were germinated on wetted filter paper at 4 °C for 48 h before transferring to a growth cabinet maintained with 16 h of light (180 μmol m^2^ s^–1^), and 8 h of dark (18 °C/16 °C). After 2 days, germinated wheat seedlings were then transferred into hydroponic growth media for safener induction treatments or into compost (John Innes No. 2) for herbicide tolerance studies. Blackgrass seedlings were germinated and maintained as described previously.^22^

Wheat cell suspension cultures were obtained from Syngenta, UK, and maintained in B5 plus vitamin media (Duchefa, NL), containing 2% (w/v) sucrose and 2.5 mg L^–1^ 2,4-D (pH 5.5). Suspension cultures were grown in 250 mL Erlenmeyer flasks containing 120 mL of media in the dark on an orbital shaker (120 rpm, 25 °C) and subcultured every 7 days. Blackgrass cell suspension cultures were initiated from coleoptile-derived callus using the procedure developed for *Brachypodium distachyon*.^57^ Blackgrass suspension cultures were grown in 250 mL Erlenmeyer flasks containing 120 mL of Linsmaier & Skoog (LS) media containing 3% (w/v) maltose, 7.5 mg L^–1^ 2,4-D, and 0.75 mg L^–1^ kinetin adjusted to pH 5.7. Cultures were maintained in the dark on an orbital shaker (120 rpm) at 25 °C and subcultured every 10 days.

### Herbicide Safening and Metabolism Studies

Seedlings of wheat were grown in compost (John Innes No. 2) to the 2-leaf stage and to the 3–5 leaf stage for blackgrass, as detailed previously.^22^ Plants were sprayed by hand to ″runoff” with treatments formulated with the surfactant Biopower (0.05% v/v). Replicated treatments consisted of 25 plants, comprising solvent controls (0.1% (v/v) acetone) and either 200 μM cloquintocet-mexyl (Syngenta, UK) ± 40 μM pinoxaden (Sigma-Aldrich, UK), 200 μM cloquintocet-mexyl ± 100 μM chlorotoluron, or 200 μM cloquintocet-mexyl ± 70 μM clodinafop-propargyl. After 14 days, herbicide damage was assessed by a combination of digital photography and determining foliar fresh biomass (FM) and plant height.

For herbicide metabolism studies with pinoxaden, the foliage of five plants was harvested (*n* = 5) 24 h after treatment and snap-frozen in liquid nitrogen. Finely ground frozen tissue (100 mg) was homogenized for 60 s in 500 μL of 80% (v/v) methanol (MeOH) at 6500 rpm (Precellus Evolution), then sonicated on ice for 15 min before centrifuging (16,000*g*, 30 min, 4 °C). After decanting the supernatant, the pellet was re-extracted with 250 μL of 80% (v/v) MeOH before recentrifuging. The combined supernatant was evaporated under vacuum at 30 °C, and the residue resuspended in 400 μL of 80% (v/v) MeOH and recentrifuged (16,000*g*, 15 min, 4 °C), prior to injecting a 2 μL sample onto a CSH-C18 (2.1 × 100 mm) column (Waters, UK) at a flow rate of 0.4 mL min^–1.^ Elution consisted of a gradient starting at 95% A (0.1% formic acid) and 5% B (acetonitrile containing 0.1% (v/v) formic acid), increasing to 95% B over 14 min. The eluent was analyzed by ultrahigh-performance liquid chromatography coupled with mass spectrometry (UPLC/MS, Xevo ACQUITY UPLC TQ-S, Waters, UK) using electrospray ionization (ESI) with collision energy ramped from 4 to 20 V to induce fragmentation. Parent cloquintocet-mexyl and pinoxaden, along with their respective metabolites, were identified from their reported fragment ions,^58^ with the metabolites quantified from calibration standard curves using authentic standards (Sigma-Aldrich).

### RNA-Seq Safener-Induction Studies

Hydroponically grown 7-day-old seedlings were treated with 20 μM cloquintocet-mexyl or the respective solvent control (0.1% (v/v) DMSO). After a 1-h treatment, they were transferred to fresh growth media and then sampled at 1, 3, 6, and 22 h. At each sampling interval, 5 seedlings were harvested, with four biological replicates (*n* = 4) used per time point. The seedlings were frozen in liquid nitrogen and kept at −80 °C until analyzed. For suspension culture treatments, wheat and blackgrass cells were used 4 days after subculture, with 0.1% (v/v) DMSO ± 20 μM cloquintocet-mexyl added. Cultures (100 mL) were harvested (*n* = 4) by vacuum filtration at 1 and 6 h after treatment and immediately frozen in liquid nitrogen. For each replicate, RNA was prepared, sequenced, and subjected to global differential gene expression analysis, essentially as described previously.^20^

### Phylogenetic Analysis

Amino acid sequences of wheat CYPs identified from the RNA-seq studies were aligned with selected amino acid sequences from the 45 wheat CYP families previously described^32^ using SEAVIEW software version 4.6.4^59^ and Muscle^60^ to identify members of the CYP81, CYP72, CYP709, and CYP71 families, respectively. Alignments were then then trimmed with trimAI software v.1.3 using the settings “gappyout”,^61^ as accessed through the Web server Phylemon 2.^62^ Trimmed alignments were used to infer maximum likelihood phylogenies using IQ-TREE software.^63^ Using the automatic selection mode (Bayesian Information Criterion), the model LG+F+I+G4 was selected for the phylogenies of wheat CYP families and those corresponding to the families CYP81, CYP709, and CYP71, while JTT+G4 was used for CYP72. Branch support values were calculated by an ultrafast bootstrap approximation. Phylogenetic trees were then edited with the iTOL tool (https://itol.embl.de/), as described.^64^

### Identification of CYP Families of Blackgrass Upregulated in the NTSR Population

To establish the CYP family and subfamily membership for the transcripts in blackgrass that were up-regulated in NTSR populations,^38^ Blastx was performed by searching an NCBI protein database restricted to wheat sequences.

### Quantitative Real-Time PCR

RNA (1 μg) from the samples taken for RNA-sequencing analysis was used for cDNA synthesis (iScript synthesis kit, Bio-Rad, United Kingdom). Quantitative real-time PCR was performed in a 15-μL reaction mix containing 300 ng of cDNA, 7.5 μL of LightCycler FastStart DNA Master SYBR Green I (Roche, United Kingdom), and 0.4 μM forward and reverse gene-specific primers, respectively. Reactions were performed on a LightCycler 96 Real-Time PCR System (Roche Diagnostics, UK) in a three-step program, including melting curve analysis: preincubation at 95 °C, amplification for 45 cycles (95 °C for 10 s, 60 °C for 10 s, and 72 °C for 20 s), followed by melting analysis (65–95 °C). Primers were designed by alignment of DNA sequences from members of the same CYP family using gene-specific 3′ sequences. Absolute quantification of transcript abundance was determined from standard curves prepared from CYP-specific double-stranded DNA fragments (gBlock, IDT, Coralville, IA, USA). Using the standard curve, Cq could then be converted to DNA copy number, and the mean absolute quantity (number of transcripts per ng mRNA) ± SD was calculated. The CYP-specific primers used are listed in Table S1.

### Proteomics Analysis of Wheat Seedlings and Suspension Cultures

Wheat 7-day-old seedlings, or 4-day-old suspension cultures were treated for 24 h with 0.1% (v/v) acetone or 20 μM cloquintocet-mexyl, then harvested, flash-frozen in liquid nitrogen, and stored at −80 °C. Approximately 10 g of seedlings or 15 g of cell cultures were pulverized in liquid nitrogen, and the resulting powder was extracted in 25 mM Tris-HCl (pH 7.6), containing 150 mM NaCl, 1% NP-40, 2% sodium deoxycholate, and 0.1% SDS. Samples were centrifuged (10,000*g*, 15 min, 4 °C) and the microsomal fraction prepared from the supernatant by ultracentrifugation (100,000*g*, 90 min). The resulting pellet was resuspended in 10 mM Tris (pH 8) buffer containing 0.1 mM EDTA and 20% glycerol. After normalizing, 15 μg of protein was loaded onto a NuPAGE gel (12% acrylamide), and the polypeptides resolved by electrophoresis at 150 V. The gel was fixed, stained with Colloidal Coomassie Blue stain, and visualized bands of interest were excised for in-gel trypsin digestion as detailed in the Supplementary Methods.

### Functional Characterization of Recombinant CYPs

CYP sequences from winter wheat or blackgrass were optimized for expression in *Saccharomyces cerevisiae* and synthesized as double-stranded DNA fragments (gBlocks, IDT, Coralville, IA, USA), prior to in-fusion cloning (Clontech, Saint-Germain-en-Laye, France) into the pYES3 vector (Thermo Fisher Scientific, Loughborough, UK). Plasmids were then transformed into WAT11 cell lines of *S. cerevisiae*,^20^ which express the CYP reductase from *Arabidopsis thaliana*. Microsomes isolated from recombinant yeast samples were prepared and quantified for recombinant CYP (rCYP) content by HPLC-MS-based proteomics, using the *N*-terminal peptide TAG *ASWSHPQFEK* for calibration.^20^ Microsomal preparations and whole-cell preparations were assayed for rCYP activity toward herbicides, and reaction products were identified and quantified by HPLC-MS as described previously,^20^ having confirmed that activity was strictly dependent on protein content and reaction time and not limited by NADPH availability.

### Statistical Analysis

One-way ANOVA followed by Tukey’s honestly significant difference (HSD) posthoc test, or the Kruskal–Wallis test followed by the Dunn test, was performed using the functions in R (stats 3.6.2 and multicomp 1.4–15 packages, respectively). ANOVA’s normality and homoscedasticity assumptions were checked in all cases using the R functions Shapiro test and Levene’s test (stats and car 3.0–10 packages, respectively).

## Supporting Information Available

Method S1: proteomic analysis; Method S2: criteria for selection of CYPs for functional characterization; Table S1: gene specific primer sequences used for the absolute quantification of transcript abundance of *Ta*CYPs by real-time qPCR; Table S2: spreadsheet showing the identification of all unigenes identified in the transcriptomic studies carried out in wheat seedlings and the fold change in their abundance resulting from a treatment with the safener cloquintocet-mexyl; Table S3: spreadsheet showing the identification of all unigenes identified in the transcriptomic studies carried out in wheat suspension cultures and the fold change in their abundance resulting from a treatment with the safener cloquintocet-mexyl. Table S4: *Ta*CYPs selected for functional characterization based on phylogenetic analysis and data mining; Table S5: levels of CYP protein present in microsome preparations from recombinant yeast expressing the respective *Ta*CYP; Table S6: CYPs upregulated in NTSR vs HS blackgrass; Figure S1: effect of treatment of wheat seedlings with pinoxaden, ± cloquintocet-mexyl; Figure S2: effect of treatment of wheat seedlings with chlorotoluron, ± cloquintocet-mexyl; Figure S3: effect of treatment with pinoxaden ± cloquintocet-mexyl on HR and NTSR blackgrass; Figure S4: the effect of treatment with clodinafop-propargyl ± cloquintocet-mexyl on wheat and blackgrass. Figure S5: phylogenetic analysis of the wheat *Ta*CYP71 family; Figure S6: phylogenetic analysis of the wheat *Ta*CYP72 family; Figure S7: phylogenetic analysis of the wheat *Ta*CYP709s family; Figure S8: schematic showing the experimental design used for proteomic analysis (PDF)
Single reference IDs, stringtie annotation, values for cloquintocet 1 h vs DMSO 1 h, cloquintocet 3 h vs DMSO 3 h, cloquintocet 6 h vs DMSO 6 h, and cloquintocet 22 h vs DMSO 22 h (XLSX)
Values for cloquintocet 1 h vs DMSO 1 h up, cloquintocet 6 h vs DMSO 6 h up, cloquintocet 1 h vs DMSO 1 h down, and cloquintocet 6 h vs DMSO 6 h up (XLSX)
