# Supplementary material for: Functional Characterization of Cytochromes P450 Linked to Herbicide Detoxification and Selectivity in Winter Wheat and the Problem Competing Weed Blackgrass
Source: ACS Omega. 2025 Mar 21;10(12):12270–87. doi: 10.1021/acsomega.4c11069 (PMC11966285; doi:10.1021/acsomega.4c11069)
Supplement: Supplementary file 1 — ao4c11069_si_001.pdf [file ao4c11069_si_001.pdf]

Supplemental Information

Functional characterization of cytochromes P450 linked to herbicide detoxification and selectivity in winter wheat and the problem competing weed blackgrass

Alina Goldberg-Cavalleri[#1], Sara Franco-Ortega[#1,3], Stewart Brown[1,6], Andrew Walker[1], Blandine Rougemont [2,4], John Sinclair[2], Melissa Brazier-Hicks[2,] Richard Dale[2], Nawaporn Onkokesung[1,5] & Robert Edwards[1]*

[1] School of Natural and Environmental Sciences, Newcastle University, Newcastle upon Tyne, NE1 7RU, UK

[2] Syngenta, Jealott's Hill, Bracknell, Berkshire, RG42 6EY, UK

[3]**Current Address**, Department of Biology, University of York, York YO10 5DD.

[4] **Current Address**, Inoviv, Hogarth House, 136 High Holborn, London, WC1V 6PX

[5] **Current Address**, Resistance Research, Bayer Aktiengesellschaft, Industriepark Höchst, H872, 65929, Frankfurt am Main, Germany

[6] **Current Address**, Enviresearch, Waterloo Square, Newcastle Upon Tyne, NE1 4DP.

[#] These authors contributed equally to the work described

***Corresponding author**, email robert.edwards@ncl.ac.uk

Supplemental methods

Proteomic analysis

Analysis of the tryptic digests derived from membrane-associated polypeptides resolved by SDS-PAGE was carried out by a methodology based on a published procedure[1]. Acidified tryptic digests were resuspended in 0.1 % formic acid and analysed on a Fusion Lumos instrument (ThermoFisher) coupled to an Ultimate 3000 liquid chromatography system (Thermo Fisher Scientific). Samples were separated on an EasySpray 50 cm, ID 75 µm (PepMap RSLC, C18, 100 Å, 2 µm particle-size) column, maintained at 50 °C using a linear gradient of 8-40 % acetonitrile at a flow rate of 300 nl min^{-1} over 120 min. Peptide ions were further separated by High field asymmetric-waveform ion-mobility spectrometry (FAIMS) (ThermoFisher) at a CV of -40 on a Fusion Lumos mass spectrometer[1], following electrospray ionization (spray voltage = 2.2 kV, S-lens RF level at 30, with heated capillary at 300°C). Full MS resolution was set to 120,000 at m/z 200 and full MS AGC target set to 5E5 using an injection time of 20 ms and a mass range set to 350-1650 m/z. After setting the AGC target value for fragment spectra to 1E6, a total of 40 variable windows were employed with a resolution set to 30,000, an injection time set to 60 ms, and a normalised collision energy set at 30%. All scan events were acquired in profile mode over 150 min. DIA raw files were processed in Spectronaut with the default settings for direct DIA, data filtering by Qvalue, and automatic normalization applied. Peptides were identified using the Uniprot *Triticum aestivum* CS ref proteome (downloaded 08062021, 130,673 entries), with Zt used for database searching. The data matrix was imported into Perseus version 1.6.13.0 and Log_2 transformed (Tyanova et al., 2016). Treated and control samples were defined, and Student's *t*-test performed generating a T-test p-value and a difference between the means. A permutation-based FDR was applied to adjust for multiple hypothesis testing.

Criteria for selection of CYPs for functional characterisation

In addition to characterising the wheat CYPs which were safener-inducible in both seedlings and cell cultures, an additional 12 family members were selected for functional characterisation based on the following criteria. *Ta*CYP81-7 was included as it formed a monophyletic group with the ortholog *Ep*CYP81A15 from *Echinocloa phyllopogon* which is linked to tolerance to the herbicide clomazone[2]. *Ta*CYP81-6 was also selected to include family 81 members not previously associated with xenobiotic metabolism (*Ta*CYP81-6 belonging to the subfamily N while the other *Ta*CYP81s belong to subfamily A). The related *Ta*CYP71-11, *Ta*CYP71-15 and *Ta*CYP71-17 genes from family 71 were included, since *Ta*CYP71-11 clusters with *Os*CYP71Ak1, which is up regulated by safeners in rice[3], while *Ta*CYP71-15 and *Ta*CYP71-17 formed a monophyletic group with maize *Zm*CYP71C3v12 (Figure 4; family 71), a gene whose expression is induced by exposure to naphthalic anhydride and triasulfuron[4.] *Ta*CYP72-7 was included as it forms a monophyletic group with maize *Zm*CYP72A354 which is upregulated by the safener metcamifen[5] From the family 709, *Ta*CYP709-4, along with *Ta*CYP709-8 and 12 were selected as orthologs of *Os*CYP709C11, which are up-regulated by fenclorim in rice, or by other safeners in wheat[3]. To include a member of other families that have not been linked to herbicide detoxification previously but which were enhanced in safener-treated wheat seedlings, one CYP from the family 94, *Ta*CYP94-1 was also included.

Supplemental Tables

Table S1. Gene specific primer sequences used for the absolute quantification of transcript abundance of *Ta*CYPs by real-time qPCR

Gene	Primer name	Sequence
TaCYP81-1	TaCYP81-1-F	5’-CTGCATCGTCTCATCGACAA-3’
	TaCYP81-1-R	5’-TAAATTCGCGCAGAGAGCCG-3’
TaCYP81-2	TaCYP81-2-F	5’-AGCAGGAGGGACGCCTTCA-3’
	TaCYP81-2-R	5’-ACAAATTCGCACAGAGAGCCA-3’
TaCYP81-3	TaCYP81-3-F	5’-GCATGGTCACCGCCGAT-3’
	TaCYP81-3-R	5’-TCTGGCCGGAACGCCGT-3’
TaCYP81-4	TaCYP81-4-F	5’-GGAACGGGTGGACGGCT-3’
	TaCYP81-4-R	5’- GCCTCCAAAGCCACGGA-3’
TaCYP81-6	TaCYP81-6-F	5’-AGTCGGACATCACCAACCTA-3’
	TaCYP81-6-R	5’- GGCGCATCCCAGATTTTGACG-3’
TaCYP81-7	TaCYP81-7-F	5’-TATGGTTCTTGGCACGCTCA-3’
	TaCYP81-7-R	5’-CTTGTGAAGAAGAGTTTCCATGC-3’
TaCYP71-15	TaCYP71-15-F	5’- GACTACTTCCCGAGCTTGG-3’
	TaCYP71-11-F	5’-GTCTCTGTCTTGATCTTGCTC-3’
TaCYP71-11	TaCYP71-11-R	5’-CAATCGACCACGATGGCG-3’
	TaCYP71-15-F	5’-CGTAGGTCCACGCCCTCA-3’
TaCYP71-17	TaCYP71-17-F	5’-TGAGCTAGAGCAACGCCAAG-3’
	TaCYP71-17-R	5’-TGGATGACGTGTCTGTGCCA-3’
TaCYP709-4	TaCYP709-4-F	5’-GCCGGTTCGAGTTCGAGG-3’
	TaCYP709-4-R	5’-AGGAGCTTGAGCAGCAC-3’
TaCYP709-5	TaCYP709-5-F	5’-GAGAACAAAGACACCATGGGT-3’
	TaCYP709-5-R	5’-TGAGCTGGTGTCATGCCCT-3’
TaCYP709-6	TaCYP709-6-F	5’-CAACGTGCAAATCCCAGCATTA -3’
	TaCYP709-6-R	5’-TCTGCCGCGCACGCCTCT-3’
TaCYP709-7	TaCYP709-7-F	5’-GCAGAGGGAGCTCCAATTC-3’
	TaCYP709-7-R	5’-TCAGCATGGTCCTCACCTCT-3’
TaCYP72-1	TaCYP72-1-F	5’-GCAAGCTATTCAGGAAGGA-3’
	TaCYP72-1-R	5’-CTCCTCCATGACTTCATCTGA-3’
TaCYP72-2	TaCYP72-2-F	5’-TGACTTACTCGGCTTATTACTC-3’
	TaCYP72-2-R	5’-CAATATTGATGTTGTCTCCA -3’
TaCYP72-4	TaCYP72-4-F	5’-CTGTTGCGAAGAAATGATTACAAG-3’
	TaCYP72-4-R	5’-CTCATGATTTGCAAGCCCGTT-3’
TaCYP72-6	TaCYP72-6-F	5’-CGGGCTTGCAAATCATGAA-3’
	TaCYP72-6-R	5’-CTTGTAATCATCTCTTCACAGCAA-3’
TaCYP72-7	TaCYP72-7-F	5’-CGGAGCGAATTATCAAGGCC-3’
	TaCYP72-7-R	5’-TAAGCCCATCGTTCTCCATC-3’
TaCYP76-3	TaCYP76-3-F	5’-TCATTGTTACATCAGTTTAAGTGGA-3’
	TaCYP76-3-R	5’-AACAACACCTAAAGCACTCATAC-3’
TaCYP89-1	TaCYP89-1-F	5’-CACGCCATGCAGCGGC-3’
	TaCYP89-1-R	5’-CGTAGGAGTGCTCGAACGT-3’
TaCYP89-2	TaCYP89-2-F	5’-CTGCGTGATGAGTCGTCG-3’
	TaCYP89-2-R	5’-CTCGACCTGTAGATGAGAAGT-3’

Table S2. Spreadsheet showing the identification of all unigenes identified in the transcriptomic studies carried out in wheat seedlings and the fold change in their abundance resulting from a treatment with the safener cloquintocet-mexyl. See attached spreadsheet

Table S3. Spreadsheet showing the identification of all unigenes identified in the transcriptomic studies carried out in wheat suspension cultures and the fold change in their abundance resulting from a treatment with the safener cloquintocet-mexyl. See attached spreadsheet

Table S4 *Ta*CYPs selected for functional characterisation based on phylogenetic analysis and data mining. For each gene, maximal n-fold induction levels in seedlings and cell cultures following treatment with cloquintocet-mexyl as compared to the solvent control (DMSO), are shown.

Gene ID for this study	Gene ID from International Wheat Genome Sequencing Consortium (IWGSC)	Nomenclature assigned by Li et al., 2020	Expression in seedling	Expression in cell culture
TaCYP81-1	TraesCS5D02G407400	TaCYP81A51_5D	26	4.9
TaCYP81-2	TraesCS5D02G407500	TaCYP81A52_5D	45.3	5.3
TaCYP81-3	TraesCS5A02G397800	TaCYP81A53_5A	6.5	3.5
TaCYP81-4	TraesCS5D02G407300	TaCYP81A53_5D	8.6	3.5
TaCYP81-5	TraesCS5B02G402800	TaCYP81A53_5B	5.7	-
TaCYP81-6	TraesCS5D02G084300	TaCYP81N20_5D		2.1
TaCYP81-7	TraesCS2D02G261100	TaCYP81A48_2D	-	2.5
TaCYP81-8	TraesCS5A02G398000	TaCYP81A52_5A	-	5.7
TaCYP71-11	TraesCS5A02G317600	TaCYP71AK13_5A	3	-
TaCYP71-15	TraesCS5D02G484900	TaCYP71C162_5D	2.3	-
TaCYP71-17	TraesCS5B02G485000	TaCYP71C163_5B	274.4	-
TaCYP709-4	TraesCS7A02G155000	TaCYP709C47_7A	8192	-
TaCYP709-7	TraesCS2D02G157800	TaCYP709C3_2A	18.4	11.3
TaCYP709-5	TraesCS2D02G157900	TaCYP709C2_2D	36.8	22.6
TaCYP709-6	TraesCS2A02G152400	TaCYP709C50_2A	3	8.6
TaCYP709-8	TraesCS2B02G177300	TaCYP709C3_2B	59.7	-
TaCYP709-15	TraesCS2B02G177600	TaCYP709C2_2B	-	9.2
TaCYP709-12	TraesCS2D02G157600	TaCYP709C50_2D	-	8
TaCYP72-1	TraesCS1A02G348800	TaCYP72A6_U	27.9	2.8
TaCYP72-2	TraesCS1D02G351800	TaCYP72A6_1D	17.1	-
TaCYP72-4	TraesCS7D02G438600	TaCYP72A600_7D	9.8	3.7
TaCYP72-6	TraesCS7A02G449500	TaCYP72A568_3B	13	3.5
TaCYP72-7	TraesCS3D02G226000	TaCYP72A587_3D	3.2	-
TaCYP76-3	TraesCS2D02G547700	TaCYP76H36_2D	24.3	3
TaCYP89-1	TraesCS1B02G203000	TaCYP89B34_1D	4	4
TaCYP89-2	TraesCS1A02G177300	TaCYP89B34_1A	5.7	4.9
TaCYP94-1	TraesCS3D02G325500	TaCYP94B3	2.5	4.9

Table S5. Levels of CYP protein present in microsome preparations from recombinant yeast expressing the respective *Ta*CYP

CYP450s	Recombinant CYP protein in yeast microsome concentration (pmol mg^{-1} microsomal protein)
**CYP81-1**	5.26
**CYP81-2**	5.90
**CYP81-3**	2.17
**CYP81-4**	0.03
**CYP81-6**	3.72
**CYP81-7**	8.65
**CYP81-8**	0.28
**CYP71-15**	0.72
**CYP71-11**	2.82
**CYP71-17**	ND
**CYP76-3**	1.16
**CYP89-1**	4.34
**CYP89-2**	0.25
**CYP709-15**	0.41
**CYP709-12**	ND
**CYP72-1**	0.16
**CYP72-2**	0.49
**CYP72-4**	2.53
**CYP72-6**	1.99
**CYP72-7**	ND

Supplemental Table S6 CYP450s upregulated in NTSR blackgrass. The orthologues in *T.aestivum* are named base on the reference nomenclature[6]

	N fold expression	Orthologue in *T. aestivum*
AmCYP81A2	13.1	TaCYP81A53_5B
AmCYP81A4	10	TaCYP81A52_5A
AmCYP71C3	3.2	TaCYP71C168_U
AmCYP71X1	8.1	TaCYP71X40_6B
AmCYP71X2	8.8	TaCYP71X41_6D
AmCYP72A5	3.6	TaCYP72A600_7B
AmCYP72A3	2.4	TaCYP72A569_3B
AmCYP72A4	5.4	TaCYP72A600_7B
AmCYP709C1	18.4	TaCYP709C3_2D
AmCYP76H1	9.2	TaCYP76H46_7A

Supplemental Figures

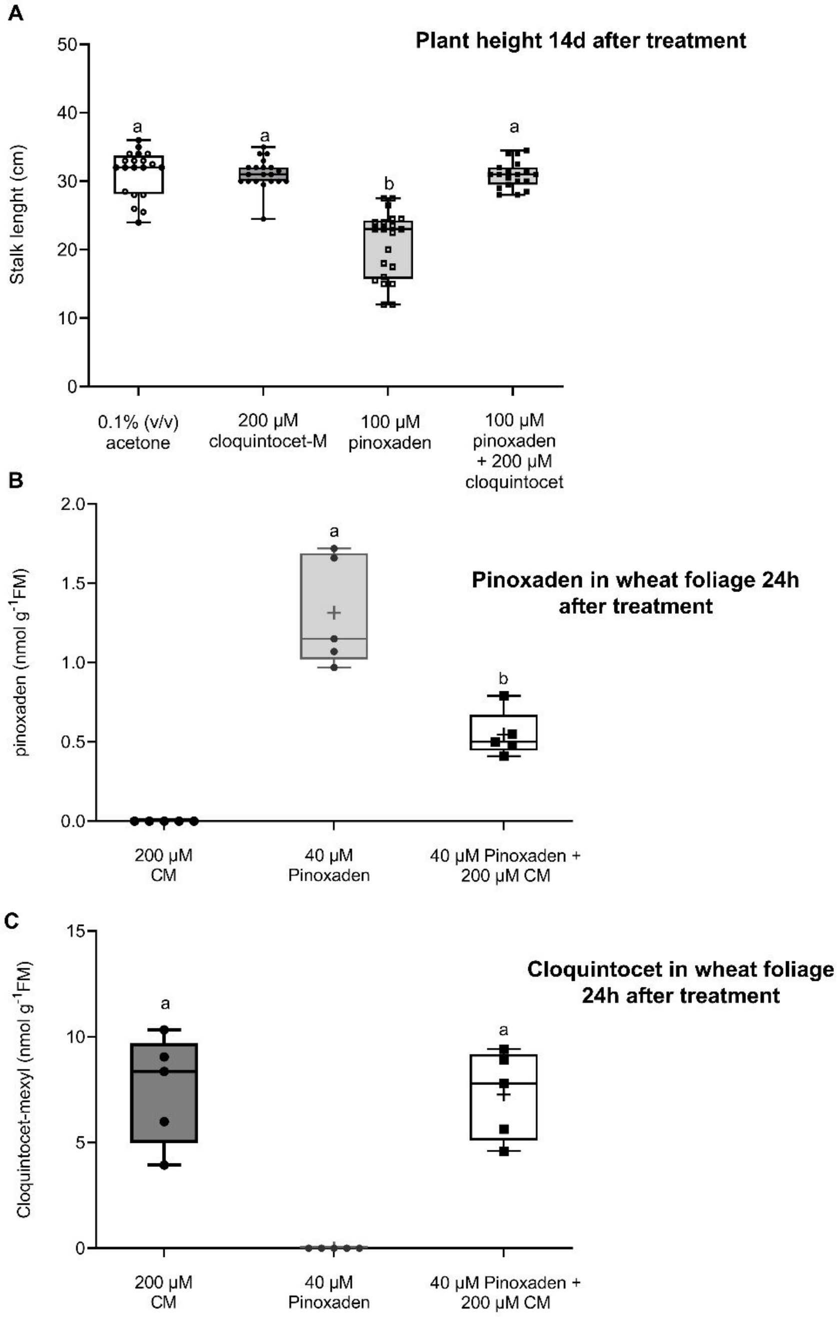

Figure S1. Treatment of wheat seedlings with pinoxaden (100 µM) ± cloquintocet-mexyl (200 µM). A. Effect of the different treatments on plant height at 14d. B. Residues of parent pinoxaden determined in plant tissue at 24 h after treatment. C. Residues of cloquintocet-mexyl determined in plant tissue at 24 h after treatment. Mean ± SD of plant height, pinoxaden concentration and cloquintocet levels from different treatments were compared by one-way ANOVA. The different letters represent significant difference among treatments (Tukey HSD post-hoc test; $p \leq 0.05$)

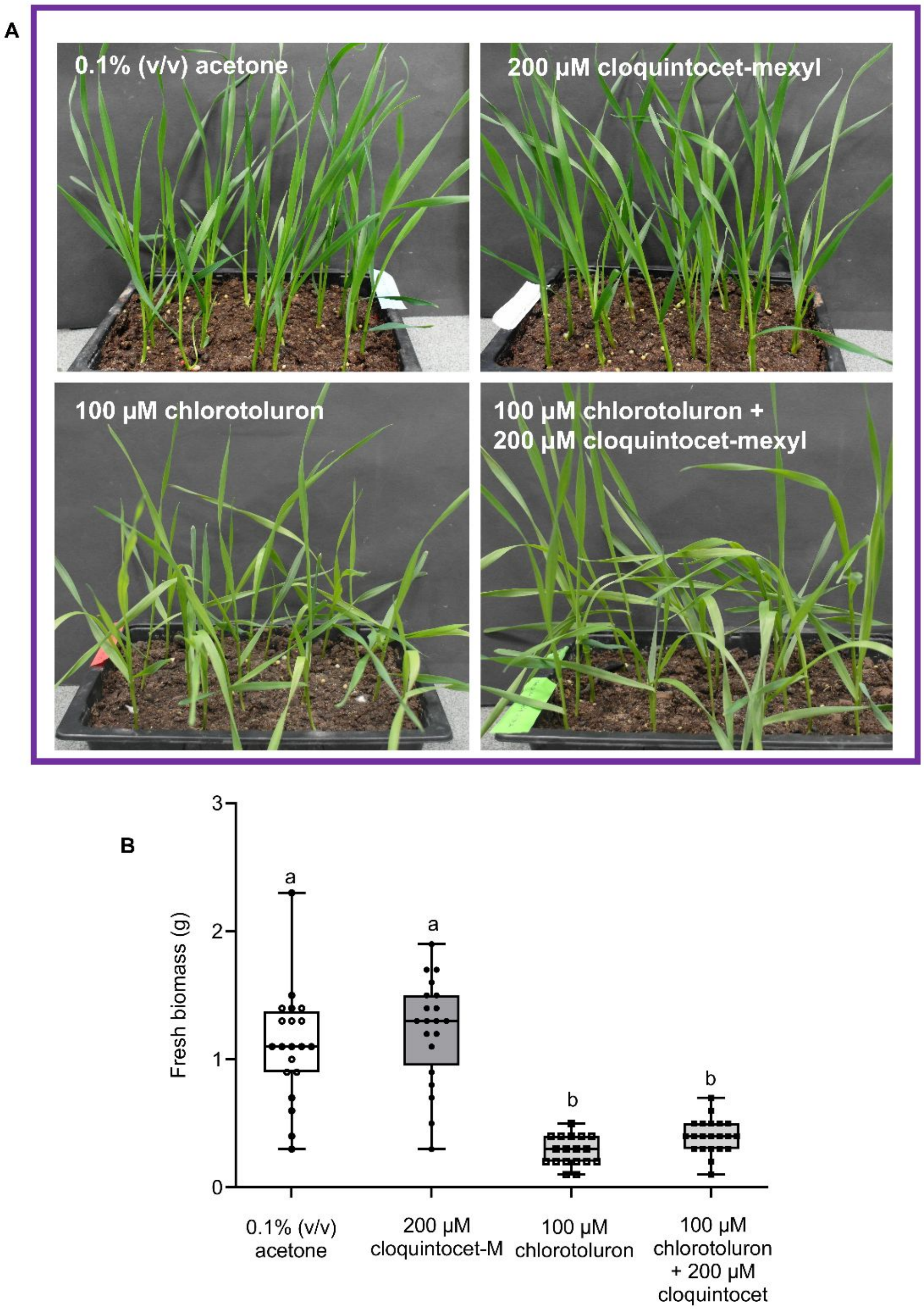

Figure S2. Treatment of wheat seedlings with chlorotoluron (100 µM) ± cloquintocet-mexyl (200 µM). A. Digital photographs of seedlings taken 14 d after treatment. B. Fresh biomass (FM) of above ground tissue determined at 14 d after treatment. FM for different treatments were compared by one-way ANOVA and the different letters represent significant difference (Tukey HSD post-hoc; $p \leq 0.05$)

HS black-grass

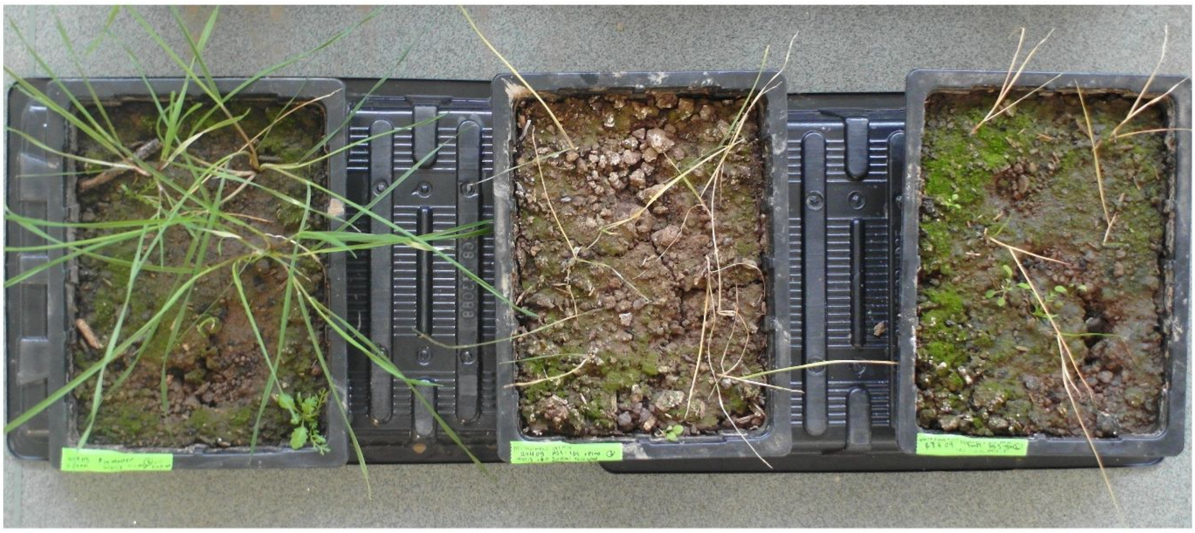

0.1% (v/v) acetone **100 μM cloquintocet-mexyl+ 40 μM pinoxaden**

NTSR black-grass

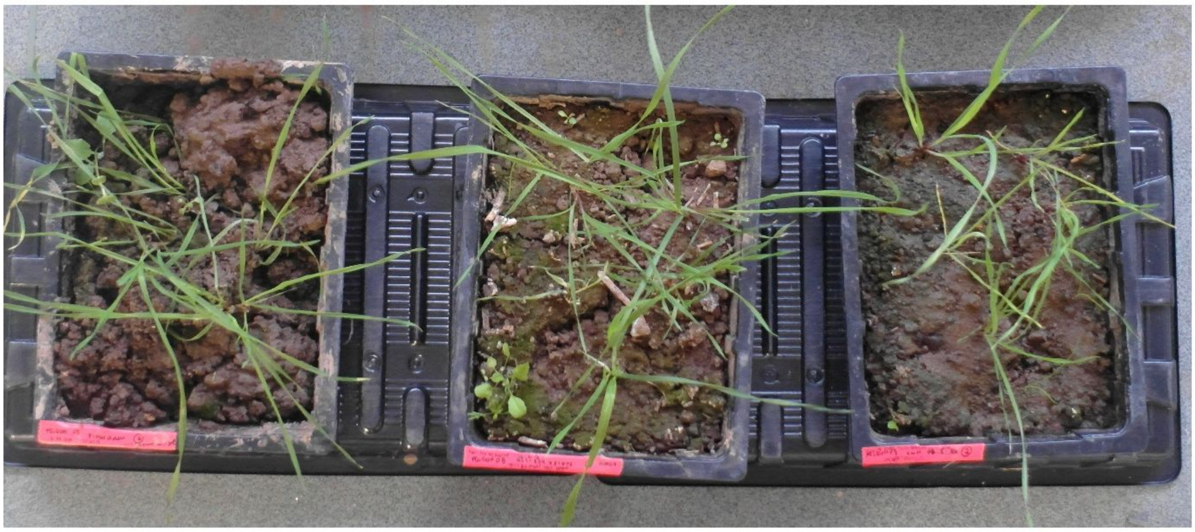

0.1% (v/v) acetone **100 μM cloquintocet-mexyl+ 40 μM pinoxaden**

Figure S3. Treatment of (A) HS, or NTSR, blackgrass populations with either control formulation (0.1% v/v acetone) or pinoxaden (40 μM) in the presence of cloquintocet-mexyl (100 μM).

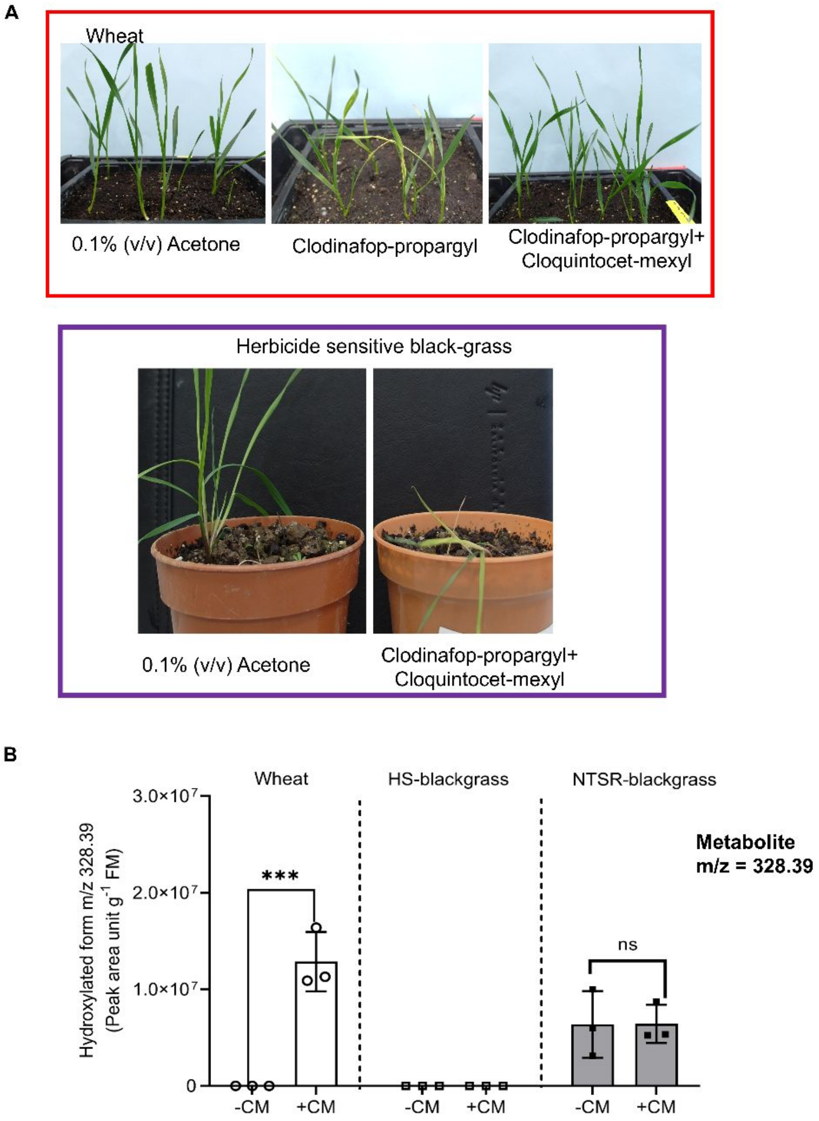

Figure S4. The different effects of herbicide exposure ± a co-treatment with the safener cloquintocet-mexyl in wheat and HS blackgrass. (A) The safening effect of cloquinocet-mexyl (100 μM) against clodinafop-progagyl (70 μM) on wheat seedlings 14d after treatment. B. The co-application of cloquinocet-mexyl (100 μM) with clodinafop-progagyl (70 μM) had no safening effect on HS black-grass at 14d after treatment. C. The level of the hydroxylated detoxified metabolite of clodinafop accumulating in cell suspension cultures of wheat, HS blackgrass and NTSR blackgrass 6 h after treatment with the 70 μM clodinafop-progagyl ± 100 μM cloquintocet mexyl. Levels of hydroxylated-clodinafop ± co-treatment with cloquintocet-mexyl in the cultures were compared within the same cell type by Student's t-test; asterisks indicate a significant difference * $p \leq 0.05$, ** $p \leq 0.01$, *** $p \leq 0.001$.

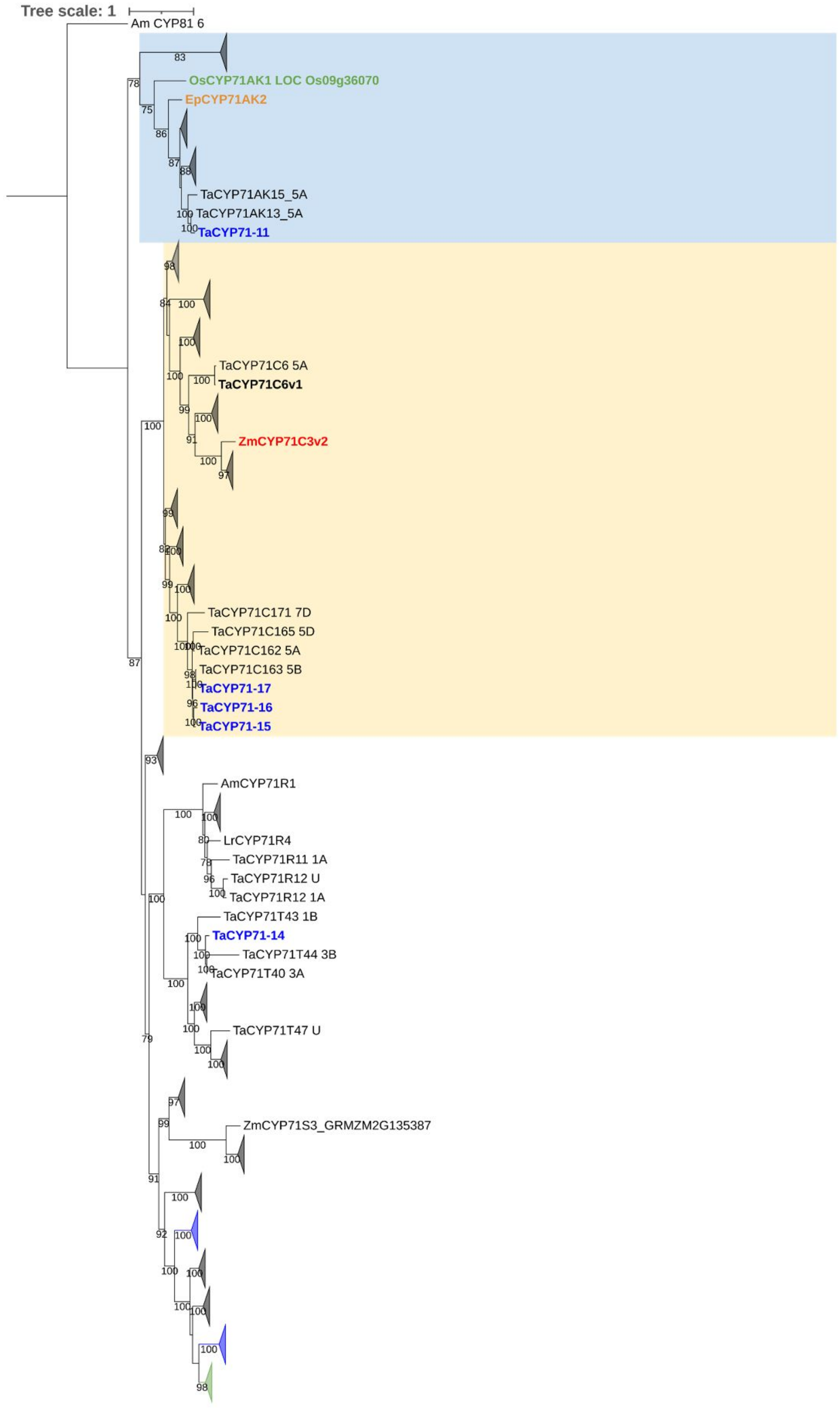

Figure S5. Phylogenetic analysis of wheat *Ta*CYP71 proteins identified in this study as referenced to related sequences linked to herbicide detoxification in wheat (*Triticum aestivum*, *Ta*), maize (*Zea mays*, *Zm*), rice (*Oryza sativa*, Os), blackgrass (*Alopecurus myosuroides*, *Am*), late watergrass

(*Echinochloa phyllopogon*, *Ep*), and annual ryegrass (*Lolium rigidum*, *Lr*). The tree is rooted using the sequence of *Am*CYP81-6, with branches with bootstrap support values above 75% labelled and the scale bar representing the inferred number of amino acid substitutions per site. Wheat sequences induced by the safener cloquintocet-mexyl are highlighted in blue text. Wheat CYPs clustering with previously identified safener -inducible CYPs from rice (*Os*CYP71Ak1)[3] and maize (*Zm*CYP71C3v12)[6] are shown against a light blue and yellow background respectively.

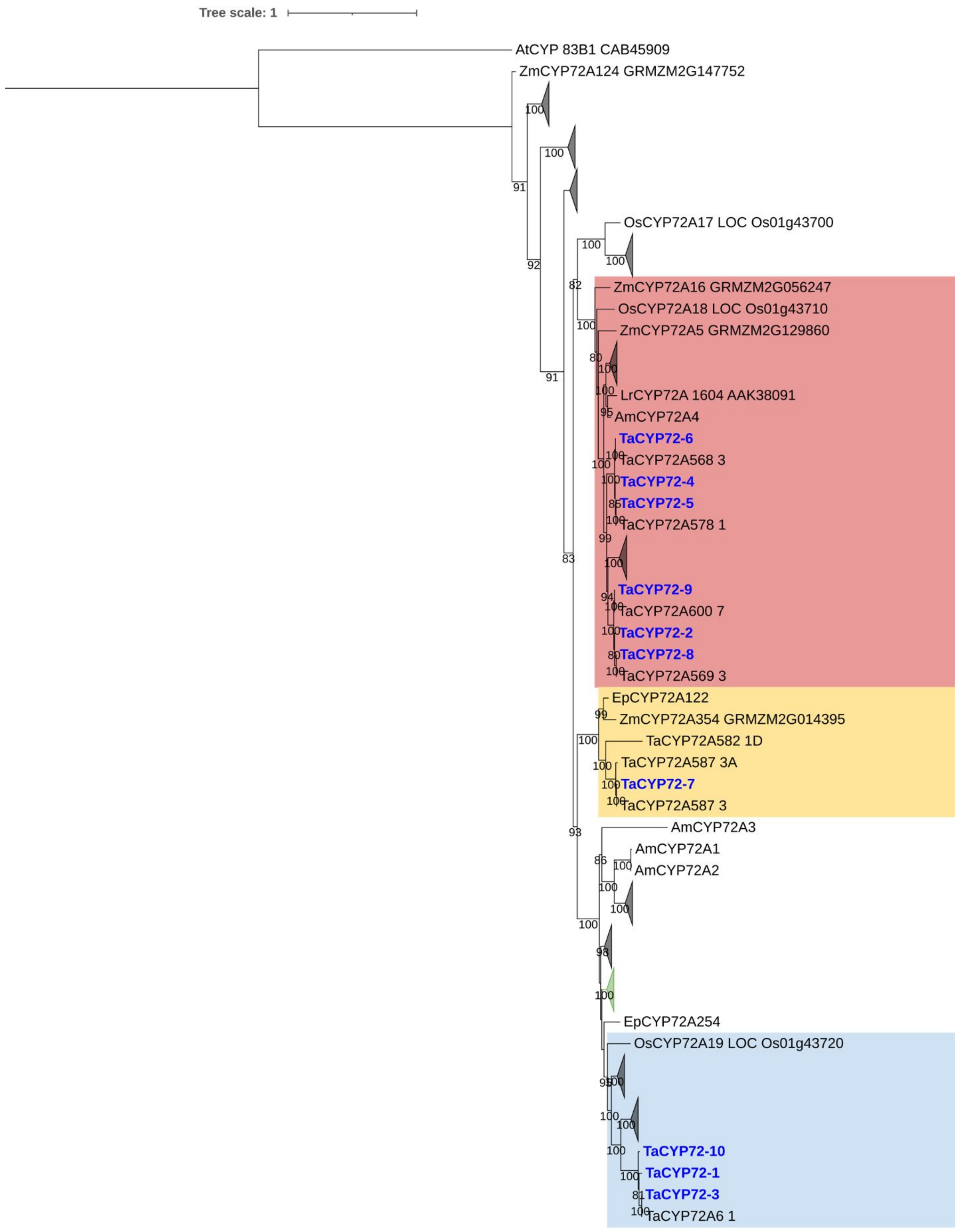

Figure S6. Phylogenetic analysis of wheat *Ta*CYP72 family proteins identified in this study as referenced to related sequences linked to herbicide detoxification in cereals and wild grasses. The

tree was rooted using the sequence of *Arabidopsis* A*t*CYP8B13, with bootstrap values above 75% shown on the branches and the scale bar indicating the number of inferred amino acid substitutions per site. Wheat sequences induced by the safener cloquintocet-mexyl are highlighted in blue text. *Ta*CYP72s clustering with safener inducible *Zm*CYP72A5 and *Zm*CYP72A16 from maize and *Os*CYP72A18 from rice are shown on a pink background, while those wheat genes clustering with the distinct clade related to the inducible maize *Zm*CYP72A354 are shown in yellow highlight.

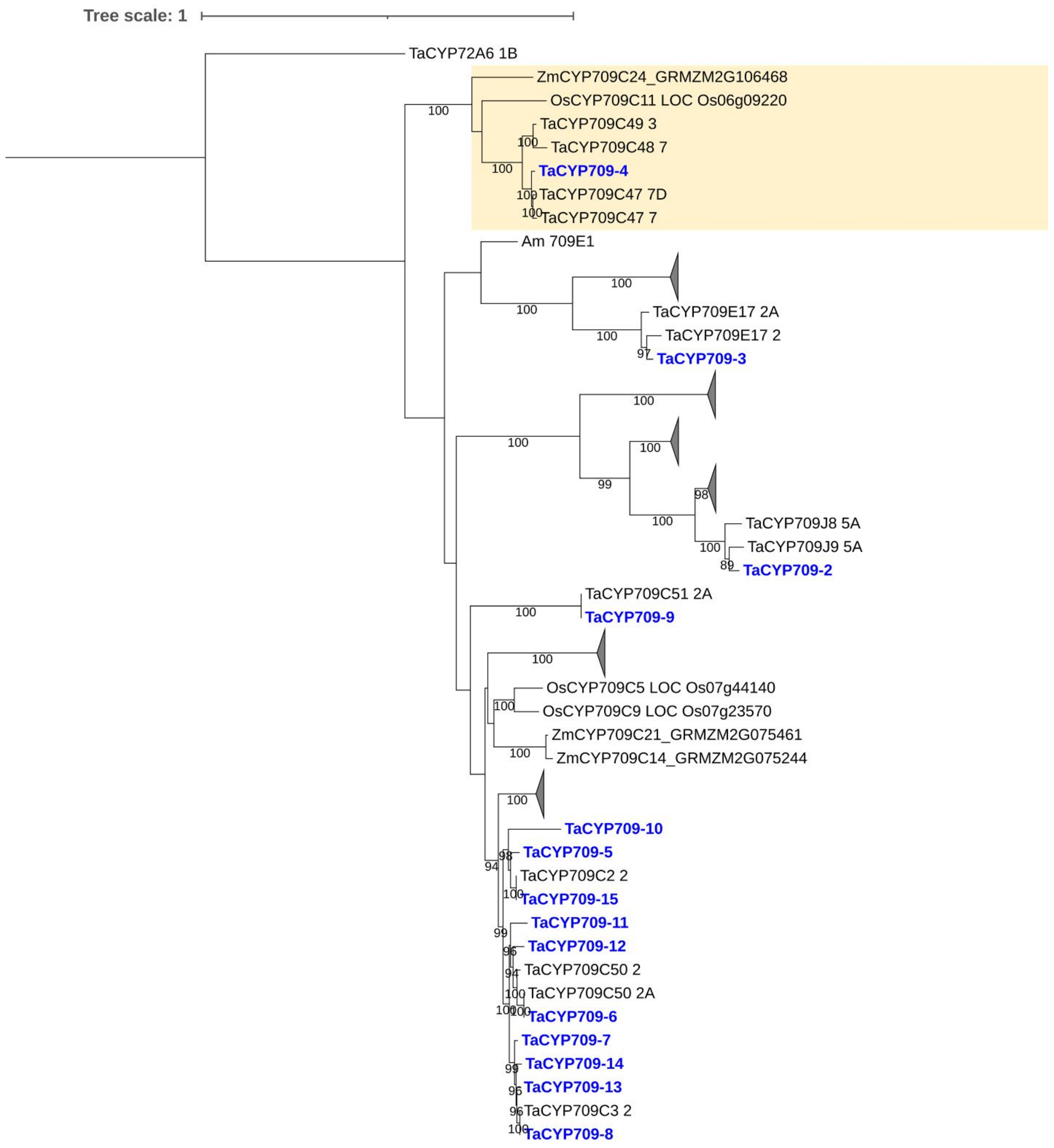

Figure S7. Phylogenetic analysis of wheat TaCYP709s family proteins identified In this study as referenced to related sequences linked to herbicide detoxification in cereals and wild grasses. The tree was rooted using the sequence of *Ta*CYP72A6 1B, with bootstrap values above 75% shown on the branches and the scale bar indicating the number of inferred substitutions per site. Wheat

sequences induced by the safener cloquintocet-mexyl are highlighted in blue text. Sequences in blue correspond to transcripts from wheat up regulated by the safener cloquintocet-mexyl. *Ta*CYP709-4 cluster with rice *Os*CYP709C11 which is up regulated by fenclorim[3]. *Ta*CYP709-8, 15 and 12 are among a monophyletic clade with exclusively wheat sequences, most of them up regulated by cloquintocet. This group is phylogenetically distant from the ones upregulated by safeners but were still selected in order to assess if they have a role in herbicide detoxification. Grey triangles: exclusively wheat sequences no modulated by cloquintocet-mexyl.

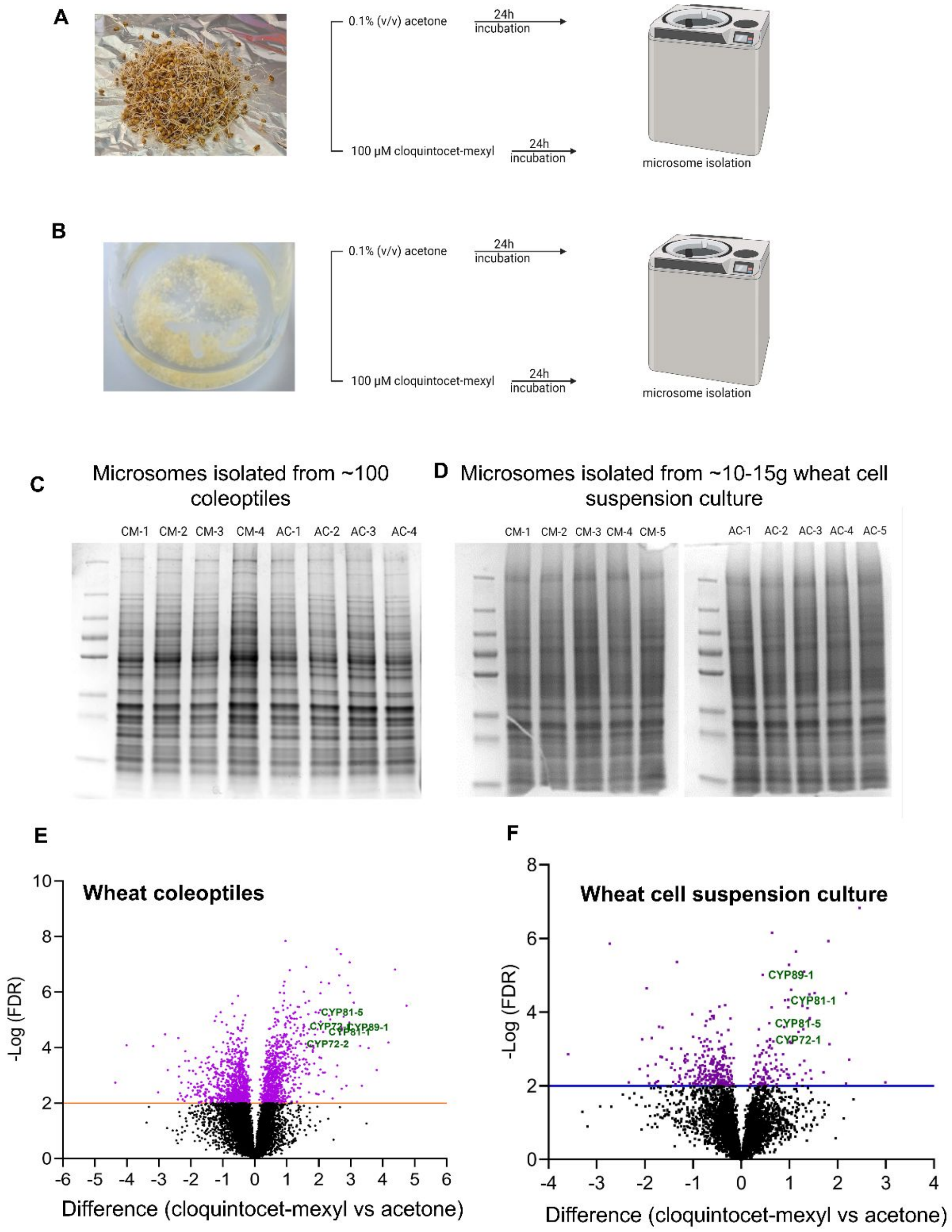

Figure S8. Schematic showing in the experimental design for proteomic analysis of (A) wheat coleoptiles and (B) suspension cultures wheat cells ± of 24 h treatment with 100 µM cloquintocet mexyl. Microsome-associated polypeptides were resolved by SDS-PAGE from (C) the coleoptiles and (D) the cell cultures, with the differential abundance of identified peptides as effected by safener treatment then determined by quantitative proteomics as represented by volcano plots for the two plant treatment systems respectively (E & F).

REFERENCES

1. Shevchenko, A., Tomas, H., Havli, J. *et al.* In-gel digestion for mass spectrometric characterization of proteins and proteomes. *Nat Protoc* **1**, 2856–2860 (2006). https://doi.org/10.1038/nprot.2006.468

2. Guo, F.; Iwakami, S.; Yamaguchi, T.; Uchino, A.; Sunohara, Y.; Matsumoto, H. Role of CYP81A cytochrome P450s in clomazone metabolism in *Echinochloa phyllopogon*. *Plant Sci.* **2019,** *283,* 321-328.

3. Brazier-Hicks, M.; Gershater, M.; Dixon, D.; Edwards, R. Substrate specificity and safener inducibility of the plant UDP-glucose-dependent family 1 glycosyltransferase super-family. *Plant Biotechnol J.* **2018**, *16*, 337-348.

4. Persans, M.W.; Wang, J.; Schuler, M.A. Characterization of maize cytochrome P450 monooxygenases induced in response to safeners and bacterial pathogens. *Plant Physiol.* **2001**, *125*, 1126-1138.

5. Brazier-Hicks, M.; Franco-Ortega, S.; Watson, P.; Rougemont, B.; Cohn, J.; Dale, R.; Hawkes, T.R.; Goldberg-Cavalleri, A.; Onkokesung, N.; Edwards, R. Characterization of cytochrome P450s with key roles in determining herbicide selectivity in maize. *ACS Omega* **2022,** *7* , 17416-17431.

6. Li, Y., Wei, K. Comparative functional genomics analysis of cytochrome P450 gene superfamily in wheat and maize. *BMC Plant Biol*. **2020,** *20,* 93.
